# *CPNE8* Promotes Gastric Cancer Metastasis by Modulating Focal Adhesion Pathway and Tumor Microenvironment

**DOI:** 10.7150/ijbs.76425

**Published:** 2022-07-25

**Authors:** Peiling Zhang, Xiaofei Cao, Mingmei Guan, Dailing Li, Hong Xiang, Qian Peng, Yun Zhou, Chengyin Weng, Xisheng Fang, Xia Liu, Haibo Mao, Qiao Li, Guolong Liu, Lin Lu

**Affiliations:** 1 Department of Medical Oncology, the Second Affiliated Hospital, School of Medicine, South China University of Technology, Guangzhou, Guangdong, China; 2 Department of Surgery University of Michigan, Ann Arbor, Michigan, USA

**Keywords:** *CPNE8*, Gastric cancer, Metastasis, Focal adhesion, Tumor microenvironment.

## Abstract

Little is known about the oncogenic role or biological function of copine Ⅷ (CPNE8) in gastric cancer (GC). Based on TCGA database, we screened for *CPNE8* and analyzed the expression of *CPNE8* in GC. The correlations between *CPNE8* and clinical features were analyzed using TCGA and GEO databases. The prognostic value of *CPNE8* was assessed using Cox analysis and Kaplan-Meier curves. The results showed that increased expression of *CPNE8* was positively correlated with metastasis and can be considered an independent prognostic risk factor for poor survival. We found that* CPNE8* can promote cell proliferation, migration, and invasiveness in GC using *in vitro* and *in vivo* experiments. Our study demonstrated that *CPNE8* promotes tumor progression via regulation of focal adhesion, and these effects can be rescued by focal adhesion kinase (*FAK*) inhibitor GSK2256098 or knockdown of *FAK*. In addition, *CPNE8* was correlated significantly with the infiltration of cancer-associated fibroblasts and immune cells, as demonstrated by various algorithms, and high *CPNE8* expression predicted poor efficacy of immune checkpoint therapy. Our findings suggest that *CPNE8* modulates focal adhesion and tumor microenvironment to promote GC progression and invasiveness and could serve as a novel prognostic biomarker in GC.

## Introduction

Gastric cancer (GC) ranks fifth in incidence and fourth in mortality worldwide 1. East Asia is among the regions with a high incidence of GC, and the incidence of GC is highest of all in China 2. Despite the advanced development of tumor-targeted therapy and immunotherapy, the prognosis of GC patients remains poor, with a 5-year overall survival rate of only approximately 30% 3-5. GC is highly susceptible to distant metastasis, leading to failure of traditional treatment strategies such as chemotherapy and radiotherapy 6. In fact, approximately 50-65% of GC patients are diagnosed at an advanced stage with extensive invasive or distant metastases, and these patients gain limited therapeutic benefit from treatment 7-9. Therefore, it is important to understand the mechanisms involved in GC progression and metastasis to contribute to the development of improved targeted therapies.

The copine family encodes calcium-dependent phospholipid-binding proteins that may play a role in membrane trafficking and in mediating cellular processes by conferring calcium regulation to various signaling pathways 10, 11. Increasing evidence has indicated that copine family members are involved in cancer development and progression. Tang summarized the biological properties of the copine family and their oncogenic roles in several cancers, including breast, colorectal, and non-small cell lung cancers 12. The copine family of proteins perform different signal transduction functions, such as membrane transport, lipid messenger production, GTPase activation, protein phosphorylation, etc. 13-15. Studies on the role of CPNEs in cancer are limited, and further research is needed. *CPNE8* (copine Ⅷ), one of the copine family genes, was initially identified as a gene pre-expressed in the prostate and testis. Robust *CPNE8* expression was detected in the prostate, heart, and brain, suggesting that *CPNE8* might play an essential role in the regulation and development of the prostate 16. CPNE family genes such as *CPNE1* and *CPNE3*, were closely related to tumorigenesis and progression 12, 17, 18. However, there is limited knowledge regarding the roles and mechanisms of *CPNE8* in carcinogenesis. *CPNE8* could fuse with *AMLI* genes to form *AML-CPNE8* chimeras in acute myeloid leukemia (AML) patients, thus negatively regulating the proliferation of AML cancer cells 19. A recent study suggested that *RP11-396F22.1*, acting as an early diagnostic indicator of cervical cancer, could enhance the progression of cervical cancer by negatively regulating *CPNE8* 20. Accumulating evidence suggests that *CPNE8* functions in cancer cell migration and invasion. Dang et al. 21 found that* CPNE8* promoted basal-like breast-cancer tumor invasion. Another study showed that* CPNE8* promoted the migration and invasion of pancreatic cancer cells 22.

In this study, we used public databases to investigate the effect of *CPNE8* on GC progression, focusing on GC metastasis. We found that *CPNE8* was indeed a metastasis-related gene. High expression of *CPNE8* in GC patients was closely associated with various clinicopathological features and predicts poor prognostic outcomes. Furthermore, we screened the downstream targets of *CPNE8* and explored their regulatory mechanisms. In addition,* CPNE8* expression is associated with multiple immune cell infiltrations in the tumor microenvironment, which may lead to GC metastasis. These findings provide a novel approach to the diagnosis and treatment of metastatic GC.

## Materials and Methods

### Characteristics of patients and ethical statement

A total of 55 patients with GC were included in this study. The patients met the diagnostic criteria for gastric adenocarcinoma, and none had received any anti-tumor treatment. All human tissues were obtained after informed consent was obtained. This study was approved by the Research Ethics Committee of the Second Affiliated Hospital of the South China University of Technology, and informed consent was obtained from all patients.

### Data collection and analysis

The mRNA data and clinical data of stomach adenocarcinoma (STAD) patients were downloaded from TCGA database and processed for differentially expressed genes and GSEA (gene-set enrichment analysis) using R scripts. Since various versions of TNM staging were applied to gastric cancer patients in the TCGA-STAD cohort, including the 5th, 6th, and 7th editions. We re-analyzed the clinical data according to the 7th edition of the AJCC TNM staging system and performed it for subsequent analyses. And the modified clinical data was shown in **Table S1**. We used the GSE118916 dataset to analyze the expression of *CPNE8* in GC. Genes associated with *CPNE8* expression were screened for KEGG the Kyoto Encyclopedia of Genes and Genomes (KEGG) and Gene Ontology (GO) for functional enrichment analysis. Statistical significance was set at *p* <0.05.

### Cell culture and transfection

The gastric mucosal epithelial cell line (GES-1) and GC cell lines AGS, BGC823, and MKN45 were obtained from the Global Bioresource Center (ATCC, USA). All cells were cultured in a DMEM medium (Gibco, USA) containing 10% fetal bovine serum (FBS) and 1% penicillin/streptomycin in a humidified atmosphere at 37 °C and 5% CO_2_. The shRNA sequence for *CPNE8* (GAGCATGGCTAGATTGGCTAA, sh-*CPNE8*) was designed and synthesized by IGE Biotechnology (Guangzhou, China). An overexpression plasmid carrying the human *CPNE8* sequence (oe-*CPNE8*) was acquired from Tsingke Biotechnology (Guangzhou, China). Lentiviruses containing scrambled sequences (sh-negative) and an empty vector (oe-vector) were used as controls according to the manufacturer's instructions. GC cells were transfected with lentiviral vectors and then selected with puromycin (a resistance marker) for one week to establish stable cell lines. Transfection efficiency was analyzed by quantitative real-time PCR (qRT-PCR) and Western blotting according to the manufacturer's instructions. We used siRNA targeting *PTK2* (encoding *FAK*) to eliminate *FAK* expression for further rescue experiments. The target sequence of *PTK2* (CCGGTCGAATGATAAGGTGTA, si-*FAK*) was synthesized by Tsingke Biotechnology (Guangzhou, China), and knockdown efficiency was determined by Western blotting.

### Western blot

Whole-cell lysates were prepared by sonication in ice-cold lysis buffer (Beyotime Biotech, Shanghai, China) containing protease inhibitors at 1:100 dilution. Subsequently, total proteins were separated by electrophoresis on 10% SDS-PAGE gels. After electrophoresis, the separated protein bands were transferred to polyvinylidene fluoride (PVDF) membranes (Millipore, Cat. #IPVH00010) and blocked with 5% skim milk for 1 h at room temperature. The membranes were then treated with primary antibodies against *CPNE8* (Cat. #AF9047), *FAK* (cat. #R24277) and pY397-*FAK* (Cat. #381143), total *ERK1 /2* (Proteintech, Cat. #67170-1-Ig), phosphorylated *ERK1 /2* (Cell Signaling Technology, Cat. # 9101), and *GAPDH* (Cat. # 5174) at a dilution ratio of 1:1000, overnight at 4 °C. The membranes were then washed in TBST for 30 min and incubated with a 1:10 000 HRP-conjugated secondary antibody (Promega, Cat. #W4021; Cat. #W4011) for 1 h at room temperature. The membranes were washed with TBST for 30 min and visualized using ECL kits (Thermo Fisher, Cat. # 34096).

### Quantitative Real-Time PCR

Total RNA was isolated from the cells using TRIzol reagent (Thermo Fisher, Cat. #15596026). Reverse transcription kit (Vazyme, Cat. #R211-01) and ChamQ Universal SYBR qPCR Master Mix (Cat. #Q711) were used for quantitative PCR of the target genes according to the manufacturer's instructions. RT-qPCR primer sequences are listed in **Table S2**. *GAPDH* gene expression was used as the endogenous control. The relative expression of the target genes relative to the control was calculated according to the 2-ΔΔCT formula. Each experiment was performed in triplicate.

### Cell viability assay

Cells were seeded in 96-well plates at a density of 2×10^3^ cells/well and allowed to grow for 5 days. Each day, 10 μl of CCK-8 (Cat. # GK10001-1) was added to 100 μl of medium and incubated at 37 °C for 2 h. Absorbance was measured at 450 nm using a multifunctional microplate instrument (Biotek Cytation5, USA). All experiments were performed in triplicate.

### Colony formation

Cells were plated in 6-well plates at a density of 500 AGS cells/well and 250 BGC823 cells/well and allowed to grow for 10 days to form visible colonies. The colonies were fixed with 4% paraformaldehyde for 15 min and stained with 0.1% crystal violet for 20 min at room temperature. The colonies were then imaged, and the number of colonies counted.

### EDU staining

An EDU staining kit (Beyotime Biotech, Cat. # C0071S) was used for this assay. Stably transfected cells were inoculated at a density of 5×10^4^ cells/well in a 24-well plate and allowed to adhere overnight for EDU staining. The cells were then washed with PBS and incubated in serum-free DMEM containing 10 μM EdU for 2 h. After fixing with 4% paraformaldehyde, the cells were stained to detect proliferating capabilities, according to the manufacturer's instructions. The cells were imaged by fluorescence microscopy, and the percentage of proliferating cells was determined.

### Flow cytometry

For cell cycle assays, 1×10^6^ cells were washed twice with ice-cold PBS, resuspended in ice-cold 70% ethanol, and fixed overnight at -20 °C. Fixed cells were then centrifuged, resuspended with 0.5 mL/test (1×10^6^ cells) PI/RNase Staining Buffer (BD, Cat. #550825), and incubated for 15 min at room temperature before analysis. PE-Annexin V Apoptosis Detection Kit I ( Cat. # 559763) was used to detect apoptotic cells, according to the manufacturer's instructions. Flow cytometry was performed using a FACScan instrument (Becton Dickinson, USA), and analysis was performed using FlowJo software.

### Mass spectrometry

Sufficient tumor cells were collected, lysed, and desalted for mass spectrometry analysis. Based on the mass-to-nucleus ratio of different substances, we detected the difference in mass-to-nucleus ratio at the cellular protein level to identify and quantify the differentially expressed proteins. The label-free proteome was detected using a mass spectrometer (Thermo Fisher Obitrap Plus). Data analysis was performed using the Proteomic Discovery software (ThermoFisher) to obtain data containing Master Protein/Gene, Relative Abundance, GO/KEGG pathway, PSM, and other information for further analysis using bioinformatics methods.

### Wound healing assay

After being imaged at 0 h, cells were cultured in a 37 °C, 5% CO2 incubator for 24 h and captured under a microscope. Cells were seeded in 24-well plates and cultured to 90% confluence. After 24 h of starvation, the cells were gently scored between monolayers of cultured cells using a sterile 20 μl tip and washed twice with PBS. The widths of the original cells and scratches after cell migration were quantified using ImageJ software.

### Migration and invasion assays

Migration and invasion experiments were performed using 24-well Transwell chambers (Cat. #3422) with 8µm wells. For cell migration experiments, 0.5 × 10^5^ cells were resuspended in 200 μl serum-free medium and placed into the upper chambers. Then, 600 µl DMEM containing 10% FBS was added to the lower chambers to incubate the cells for 24 h. For cell invasion experiments, Matrigel (BD, Cat. # 356234) at a dilution of 1:8 was pre-coated on the upper chamber surface, and then 2×10^5^ cells resuspended in 200 μl serum-free medium were added to the upper chambers. DMEM (600 μl) containing 10% FBS was added to the lower chambers and incubated for 24-48 h. The cells were then removed from the surface of the upper membrane using a cotton swab and washed three times in PBS to ensure that no cells remained on the surface. The cells were fixed with methanol at room temperature for 15 min and stained with 0.1% crystal violet for 20 min. After washing with PBS, cells were examined and counted under a microscope.

### Cell adhesion assay

The 24-well plates were precoated with 10 μg/ml fibronectin (Cat. # F8180-1ml) overnight at 4 °C, and 2×10^5^ cells per well were incubated in a 37 °C, 5% CO_2_ incubator for 2 h. The plates were gently washed with PBS to remove the non-adherent cells. Adherent cells were fixed with 4% formaldehyde and stained with 0.1% crystal violet. Cells were imaged and counted under a microscope.

### Select the optimal concentration of FAK inhibitor

GC cells were cultured in a 6-well plate and allowed to reach 70% confluence in regular culture medium. The medium was then replaced with fresh medium containing 0-10 μM GSK2256098 (TargetMol, Cat. #T2281), a small-molecule *FAK* inhibitor. The cells were incubated for 2 h. At the end of the treatment, the cells were extracted to detect *FAK* expression levels using western blotting. The concentration with the most significant reduction in *FAK* protein expression was selected as the optimal concentration for subsequent experiments.

### Animal experiments

All animal experiments were performed according to the protocol approved by the Animal Care and Use Committee of South China University of Technology. Five to 6-week-old male BALB/c nude mice were purchased from Guangdong Scarjindar Biotechnology Co. For the tumor growth model, 2×10^6^ BGC823-oe-*CPNE8* cells and control cells in 200 μl PBS were injected subcutaneously into the left lower dorsum of the nude mice. Tumor volume was measured every 3 days and calculated according to the following formula: tumor volume (mm^3^) = length × width^2^ × 0.5. BGC823-oe-*CPNE8* cells or control cells (1×10^6^/100 μl PBS) were injected into the tail vein of male BALB/c nude mice to induce tumor lung metastasis. After 4 weeks, the mice were sacrificed and all tumors and lungs were surgically removed for imaging and embedding. HE staining was performed to visualize metastatic lesions.

### Immunofluorescence staining

Mouse xenograft tumors were fixed in 4% paraformaldehyde for 24 h, embedded in paraffin, and cut into 5 µm sections. Tissue sections were placed in an oven at 60 °C for 2 h, then routinely dewaxed and heated with sodium citrate buffer (0.01 M, pH 6.0) for antigen repair. After blocking the antigen with goat serum for 30 min, the tissues were incubated with the antibody Ki67 (Zen BioScience, Cat. #381101-50 μl) overnight at 4 °C. The tissues were stained with secondary antibodies (Cat. # E032620). Parallel staining with secondary antibodies was used as the control for signal specificity. DAPI (Beyotime Biotechnology, Cat. # C1005) was used to stain the nuclei.

### Immunohistochemistry staining

Tissue sections of the xenograft tumors were treated as described above. After antigen repair, endogenous peroxidase was inhibited with 3% hydrogen peroxide for 10 min, blocked with goat serum for 30 min, and incubated with the primary antibody *CPNE8* (Proteintech, Cat. #20097-1-AP), *FAK* (Cat. #R24277) and anti-α-SMA antibodies (Cat. #GB111364), respectively, at a dilution of 1:100, at 4 °C overnight. After washing with PBS, the DAB kit (Cat. #AR1027) was used for immunostaining, and hematoxylin was used for re-staining. The results were analyzed using a microscope. The immunohistochemical score was calculated as the product of the staining intensity (0-3 points) and staining area (0-3 points) by two researchers, ZPL and CXF 23. The staining intensity was scored as follows: 0 for no staining, 1 point for light yellow, 2 points for yellow, and 3 points for brown. The stained area was scored as follows: ≤5% scored 0, 5-25% scored 1, 25-50% scored 2, and ≥50% scored 3. Immunohistochemistry scores of <3 indicated a low expression group, and a score ≥3 indicated a high expression group.

### Immuno-functional analysis

To explore the association of *CPNE8* expression with the tumor microenvironment, we evaluated the immune score, estimated score (that implies tumor purity), and stromal score of GC patients using the R package “estimate” 24. We visualized the correlation between *CPNE8* expression and cancer-associated fibroblasts (CAFs) using TIMER2 25. We also analyzed the relationship between *CPNE8* and CAFs in the GSE84437 database using the R package “estimate.” Using the TISIDB database, we determined the correlation between *CPNE8* and various chemokines and chemokine receptors 26. IHC and qRT-PCR analyses were performed to verify the relationship between *CPNE8* and CAFs further. We applied the “MCP-counter” algorithm to quantify marker-based gene sets for immune infiltration 27. We used the R package “GSVA” for single-sample gene set enrichment analysis (ssGSEA), which calculates the absolute enrichment of gene sets in each sample to assess the connection between *CPNE8* and immune-related pathways. We downloaded mutation data from the TCGA-STAD cohort, calculated TMB and MSI scores, and used Spearman's correlation analysis to characterize their correlation with *CPNE8* expression. Peng et al. designed a new computational framework that integrates two previously studied tumor immune escape mechanisms and tumor immune dysfunction and exclusion (TIDE) scores 28, 29. We used the TIDE database to calculate the correlation between *CPNE8* and dysfunction and TIDE scores. In addition, we calculated and compared the correlation of *CPNE8* expression with immune checkpoint responses using the chi-squared test.

### Statistical analysis

All statistical analyses were conducted using R software (version 4.0) and GraphPad Prism package (version 8.3). The median of *CPNE8* expression was chosen as the cut-off for the high- and low-expression groups. Student's t-test or Wilcoxon test was used for comparisons between the two groups. Analysis of variance was used to compare multiple groups. Spearman analysis was used to assess linear relationships. The chi-squared test was used to analyze the correlation between *CPNE8* expression and clinicopathological features or immune responses. For survival analysis, we selected GC patients with detailed survival status and survival time, including all stages of the disease. We used univariate and multivariate analyses to establish Cox regression models and plotted survival curves using the Kaplan-Meier method, including overall survival (OS), progression-free survival (PFS), and disease-free survival (DFS). We also performed ROC analysis using the R package “pROC” to evaluate the performance of multiple genes in the prediction of tumor carcinogenesis and metastasis, as well as survival analysis 30. All experiments were repeated at least three times, and the data are expressed as mean ± standard deviation (SD). Statistical significance was defined as two-tailed *p* <0.05.

## Results

### CPNE8 expression is increased in GC and associated with poor survival outcomes

**Figure 1:**
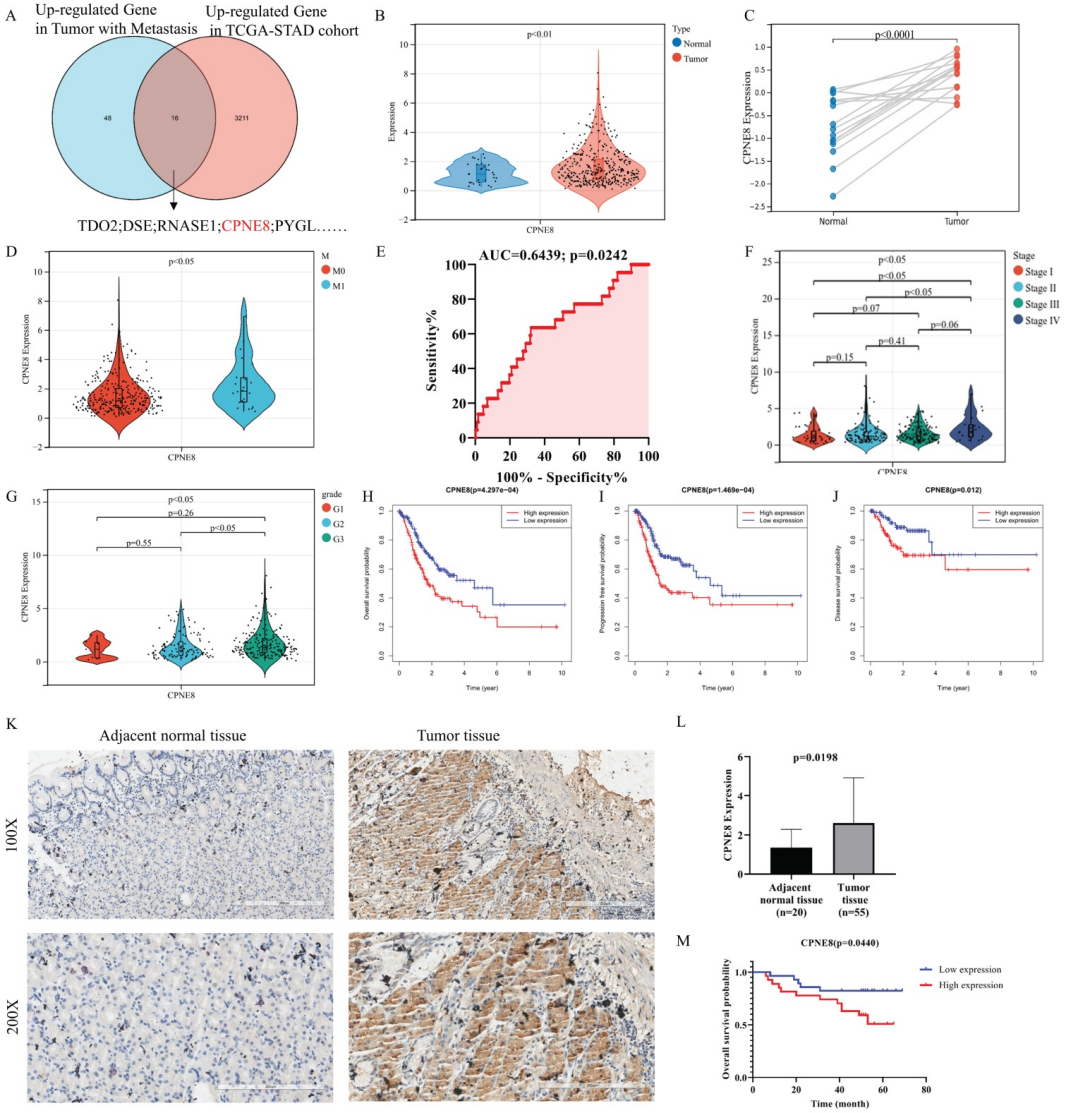
*CPNE8* was overexpressed in gastric cancer and associated with poor patient survival. (**A**) Venn diagram showing genes with high expression and association with tumor metastasis in the TCGA-STAD cohort. (**B**) Comparison of *CPNE8* gene expression between 375 tumor tissues and 32 normal tissues. (**C**) Normalized GEO data demonstrating differential expression of *CPNE8* gene between 15 tumor tissues and paired normal tissues. (**D**) Increasing *CPNE8* expression in metastatic gastric cancer compared to primary tumors. (**E**) The area under the ROC curve (AUC) indicated that *CPNE8* revealed better efficiency in predicting metastatic gastric cancer. (**F**) Significantly different expression of *CPNE8* among tumor stages in the TCGA-STAD cohort. (**G**) Significantly different expression of *CPNE8* among tumor grades in the TCGA-STAD cohort. (**H**) Overall survival (OS) curves based on *CPNE8* status. (**I**) Progression-free survival (PFS) curves based on *CPNE8* status. (**J**) Disease-free survival (DFS) curves based on *CPNE8* expression. (**K**) The representative microscopy images of *CPNE8* in normal tissue and gastric cancer tissue were shown at 100X and 200X magnifications. (**L**) The IHC scores displayed that CPNE8 was upregulated in most GC than normal tissue. (**M**) The overall survival curve was based on CPNE8 scores obtained by immunohistochemistry.

To identify candidate genes involved in GC progression and metastasis, we re-analyzed the TCGA stomach adenocarcinoma RNA-seq dataset and screened 16 genes that were highly expressed in GC and associated with metastasis, including *CPNE8* (**Figure 1A**). Most of these genes were reported to be associated with tumor metastasis, such as TDO2, CYTL1, and LDB2. 31-33. However, the role of *CPNE8* in GC oncogenesis and metastasis remains poorly understood. Therefore, we analyzed *CPNE8* mRNA levels using publicly available datasets, such as TCGA-STAD and GSE118916 cohorts. In TCGA-STAD data, *CPNE8* was upregulated in GC tissues, compared to normal tissues (**Figure 1B**, p <0.001). *CPNE8* expression was higher in tumor tissues in the GSE118916 dataset (**Figure 1C**, p <0.001). Moreover, *CPNE8* expression was upregulated in metastatic GC, compared with that in primary tumors (**Figure 1D**, p <0.05**)**, especially in patients with liver metastases (Supplementary Figure S1A, p <0.001). These results indicated that *CPNE8* was highly expressed in GC, and metastatic tumors showed higher *CPNE8* expression. To estimate the predictive value of *CPNE8* expression in gastric carcinogenesis and metastasis, we performed ROC analysis on the TCGA-STAD cohort and GSE118916 datasets. CEACAM5, encoding CEA, is usually used as a clinical biomarker for gastrointestinal cancers and may promote tumor development through its role in cell adhesion 34. The results showed that *CPNE8* was no better a predictor of the carcinogenesis of GC than CEACAM5 (Supplementary Figure S1B, *CPNE8*: AUC=0.5847, p = 0.1117; CEACAM5: AUC=0.6351, p = 0.0112) in the TCGA-STAD cohort. However, *CPNE8* exhibited excellent performance in the diagnosis of GC compared to CEACAM5 in the GSE118916 dataset (Supplementary Figure S1C, *CPNE8*: AUC=0.9422, p <0.0001; CEACAM5: AUC=0.5911, p = 0.3952). Additionally, *CPNE8* exhibited good prediction of metastatic GC (**Figure 1E**, AUC=0.6439, p =0.0242), supporting the clinical significance of *CPNE8* as an indicator of metastatic GC. Furthermore, the correlations between *CPNE8* expression and GC clinical features were analyzed (**Table 1**). The data indicated that the expression of *CPNE8* was significantly related to tumor grade (p =0.0212). As shown in **Figure 1F**, GC patients with advanced-stage exhibited higher *CPNE8* expression than those in the early stage. Likewise, high *CPNE8* expression was associated with an advanced GC grade (**Figure 1G**, p <0.05). However, there were no significant relationships between *CPNE8* expression and other clinical features such as sex, tumor invasion depth, and lymph node metastasis (Supplementary Figure S1D-F). These data suggested that high *CPNE8* expression predicts a more malignant phenotype. We further explored the predictive value of *CPNE8* in prognosis and survival of different types of patients. Univariate Cox analysis showed that *CPNE8* expression was a predictor of overall survival in GC patients (p <0.001) and remained an independent factor in multivariate Cox analysis (p =0.009) (Supplementary Figure S2A-B). Kaplan-Meier analysis indicated that higher *CPNE8* expression was significantly associated with worse overall survival (OS), progression-free survival (PFS), and disease-free survival (DFS) (p <0.01, **Figure 1H-J**). ROC curves showed a favorable predictive capacity of *CPNE8* expression for OS, PFS, and DFS in the TCGA-STAD cohort (AUC >0.5) (Supplementary Figure S2C-E). Immunohistochemistry was used to detect the expression of *CPNE8* in paraffin-embedded GC and normal tissues. **Figure 1K** shows representative microscopic images of adjacent normal tissue and GC. IHC scores showed that *CPNE8* was upregulated in most GC tissues compared to that in normal tissues (p =0.0198, **Figure 1L**). There was no significant correlation between *CPNE8* expression and TMN stage in our patient samples (data not shown). Survival analysis based on *CPNE8* expression showed that* CPNE8* was significantly associated with poorer overall survival in our patient samples (*p* =0.0440, **Figure 1M**). Taken together, these data indicate that the *CPNE8* gene was closely related to GC metastasis and is a novel prognostic biomarker that can independently predict a poor outcome in GC patients.

### CPNE8 enhances the proliferation of GC cells

**Figure 2:**
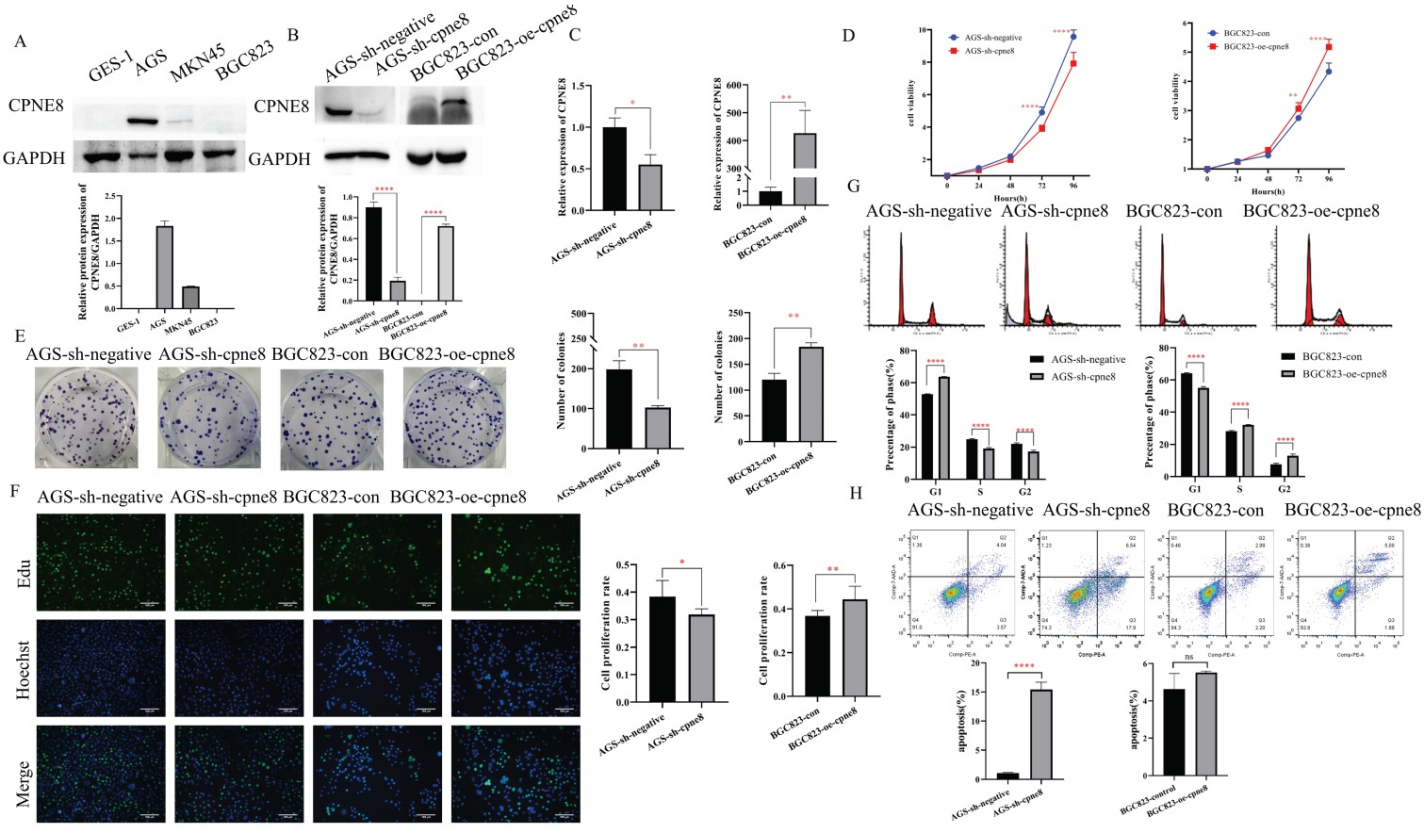
*CPNE8* enhanced the proliferation of GC cells. (**A**) Western blot plots showing the different expressions of the *CPNE8* gene in GC cell lines. Relative protein expression (*CPNE8* protein/*GAPDH*) was quantified in a column graph. (**B**) *CPNE8* knockdown and transfection efficiency in GC cells was analyzed by Western blot and quantified as relative protein expression of *CPNE8*/*GAPDH*. (**C**) The knockdown and transfection efficiency of *CPNE8* in GC cells was confirmed by qRT-PCR. The expression of mRNAs was calculated using the 2-ΔΔCt method. (**D**) CCK-8 assay showed significant differences in GC cell proliferation with knockdown or overexpression of *CPNE8*, respectively. (**E**) A colony formation assay assessed the effect of *CPNE8* on GC cell proliferation. Colonies were stained with crystal violet, and the number of colonies was counted and shown in a column diagram. Error bars represent mean ± SD from three independent experiments. (**F**) EDU staining of proliferating cells. GC cells were analyzed using a fluorescence microscope (Olympus-Microsystems). DNA (blue) was stained with Hoechst. Cyan cells show EDU/Hoechst-positive cells. The column diagram represented the proliferation rates in various GC cells. Data are presented as mean ± SD for at least three independent experiments. (**G**) Cell cycle analysis in GC cells with respective knockdown or overexpression of *CPNE8*. The bar charts represented the proportion of cells in various cell cycle phases in GC cells. Data are presented as mean ± SD for at least three independent experiments. (**H**) Analysis of apoptosis in GC cells with knockdown or overexpression of *CPNE8*. The proportion of apoptotic cells in GC cells was also shown as column graphs. Data are presented as mean ± SD for at least three independent experiments.

We performed Western blotting to detect the expression levels of *CPNE8* in the three GC cell lines. Our results showed that *CPNE8* was highly expressed in AGS cells and barely detected in BGC823 cells (**Figure 2A**). To gain insight into the effects of *CPNE8* on the cellular behavior of GC tumorigenesis, we generated the BGC823-oe-*CPNE8* cell line, which stably overexpressed full-length *CPNE8*. We also developed an AGS-sh-*CPNE8* cell line that stably expressed *CPNE8*-shRNA to suppress *CPNE8* expression. The expression levels of *CPNE8* in stably overexpressing or knocked down cell lines were confirmed by Western blot analysis (**Figure 2B**) and qRT-PCR (**Figure 2C**). Relative protein expression (*CPNE8* protein/*GAPDH*) was also quantified and is shown in a column diagram (**Figure 2B**). To determine the effect of *CPNE8* on cell proliferation, we performed a cell proliferation assay using CCK-8. We found that after stable knockdown of *CPNE8*, the proliferation ability of AGS cells was reduced, while the proliferation ability of BGC823 cells increased with the overexpression of *CPNE8* (**Figure 2D**). Consistently, knockdown of *CPNE8* significantly attenuated proliferation, whereas overexpression *CPNE8* increased the proliferation of GC cells in colony formation assays (**Figure 2E**). EDU staining assays also showed significant differences in the proliferative capacities of GC cells with *CPNE8* knockdown or overexpression compared to control cells (**Figure 2F**). Taken together, these results demonstrate that *CPNE8* promotes GC cell proliferation.

To explore the mechanism of the carcinogenesis-promoting effect of *CPNE8* on GC cells, we performed cell cycle and apoptosis analyses. Flow cytometry analysis showed an increase in the percentage of G1 cells and a decrease in the rate of S/G2 phases in AGS-sh-*CPNE8* cell, while the opposite was observed in BGC823-oe-*CPNE8* cell (**Figure 2G**). These results indicate that *CPNE8* promoted cellular G1/S phase transition. Knockdown of *CPNE8* increased the percentage of apoptotic cells, although overexpression of *CPNE8* did not significantly affect cell apoptosis (**Figure 2H**). These results suggested that *CPNE8* is involved in the cell cycle and apoptosis of GC cells.

### CPNE8 promotes GC cell migration and invasion *in vitro*

**Figure 3:**
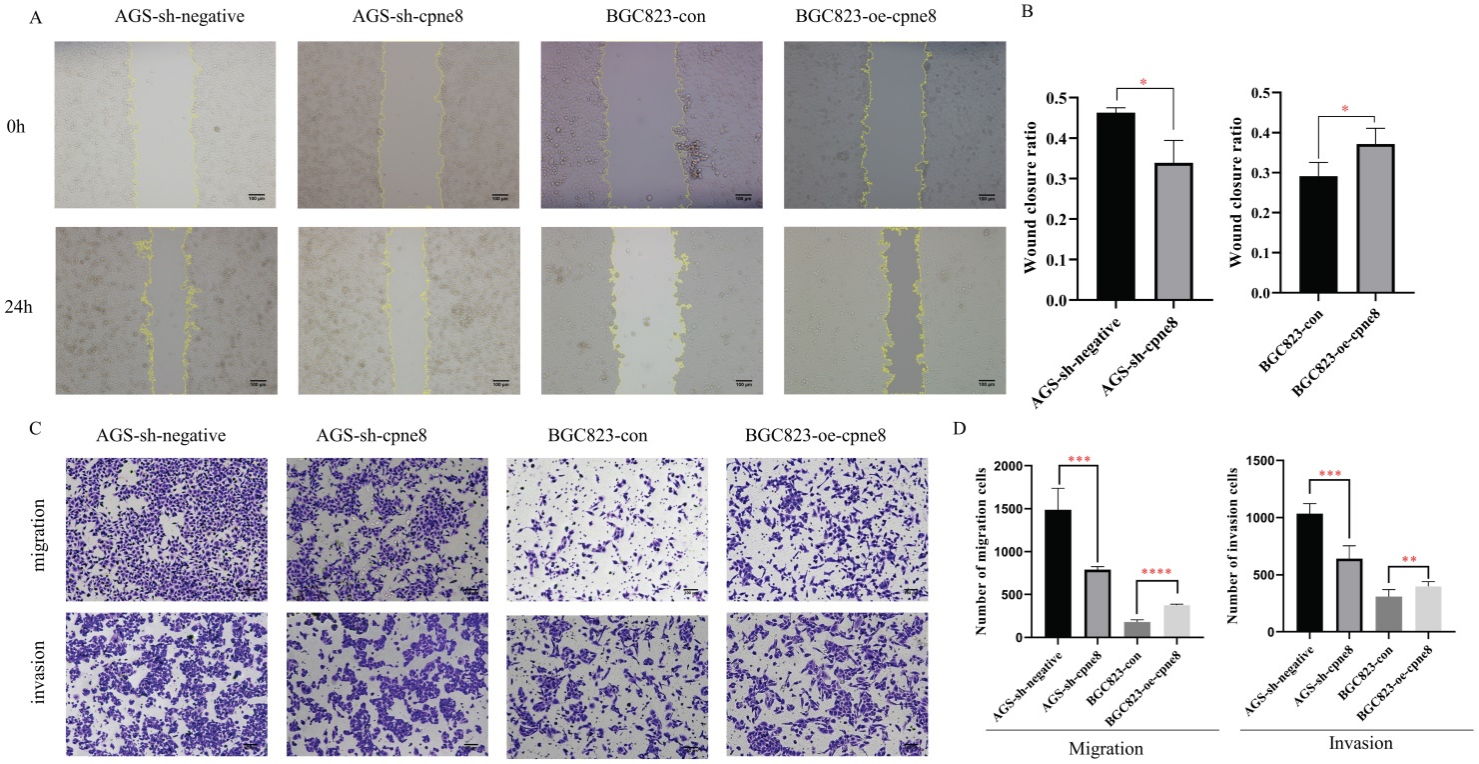
*CPNE8* promoted GC cell migration and invasion *in vitro*. (**A**) Wound-healing assays revealed that stably suppressed *CPNE8* expression inhibited migration of GC cells *in vitro*, whereas elevated *CPNE8* expression had the opposite effects. (**B**) Quantitative analysis of wound closure. (**C**) Transwell assays showed that knockdown of *CPNE8* reduced the migration and invasion of GC cells while overexpression of *CPNE8* enhanced the migration and invasion ability of the GC cells. (**D**) Quantitative analysis of migration and invasion of GC cells. Data are presented as mean values ± SD of three independent experiments.

Considering the significant elevation of *CPNE8* in metastatic GCs, we performed experiments to examine the malignant behavior of invasiveness using wound-healing and Transwell assays. Wound-healing assays demonstrated that knockdown of *CPNE8* retarded cell migration and motility. By contrast, overexpression of *CPNE8* enhanced cell migration and motility (**Figure 3A**). Quantitative analysis of wound closure area showed statistically significant differences after knockdown or overexpression of *CPNE8* compared to controls (**Figure 3B**). Transwell assays showed that migratory and invasive abilities were significantly suppressed after *CPNE8* knockdown, whereas cell migration and invasion capabilities were enhanced using BGC823-oe-*CPNE8* compared with controls (**Figure 3C**). Representative images are shown, and the numbers of migratory and invasive GC cells were quantified in a histogram graph, displaying statistically significant alterations after knockdown or overexpression of *CPNE8* (**Figure 3D**).

Collectively, these results demonstrate that *CPNE8* promotes GC cell migration and invasion *in vitro.*

### CPNE8 activates the Focal adhesion pathway to promote tumor metastasis

**Figure 4:**
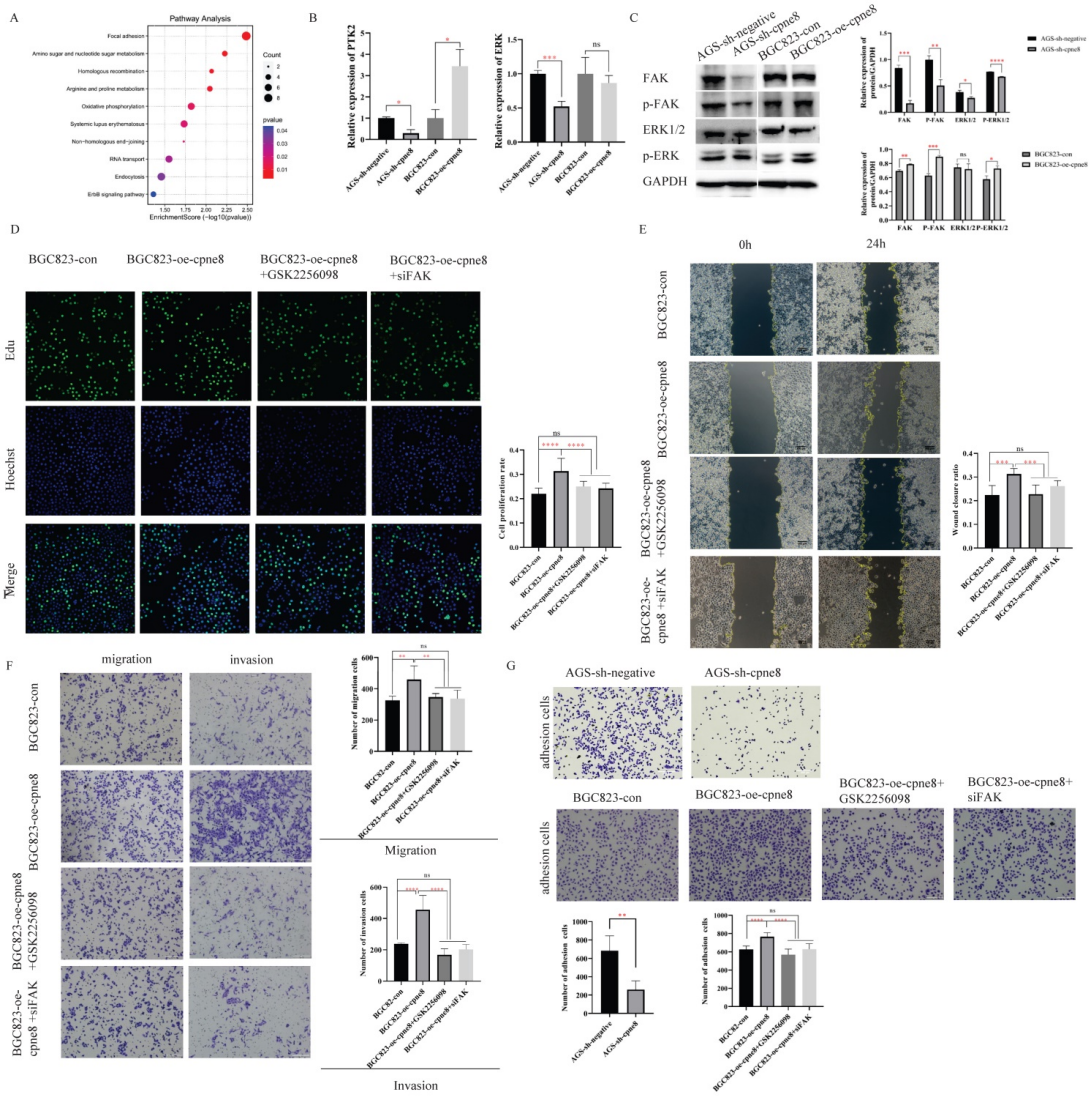
*CPNE8* acted on the Focal adhesion pathway to promote GC metastasis. (**A**) KEGG functional enrichment analysis revealed a significant correlation between *CPNE8* expression and Focal adhesion pathway in AGS-sh-*CPNE8* cells compared to the control cells. (**B**) There are relative changes of *FAK* (Gene Symbol as PTK2) and ERK mRNA levels in GC cells with knockdown or overexpression of *CPNE8*. (**C**) Western blot analysis of *FAK*, p-*FAK*, total Erk1/2, and p-ERK protein expression in GC cells with knockdown or overexpression of *CPNE8*. Relative protein expression (interested protein/*GAPDH*) was also quantified in GC cells. (D) EDU staining assay showed the proliferating cells with or without diminishing *FAK* expression, such as GSK2256098 treatment and knockdown of *FAK*. Photos were captured using a fluorescence microscope (Leica-Microsystems). DNA (blue) was stained with Hoechst. Cyan cells showed EDU/Hoechst-positive cells. The column diagram represented the proliferation rates in various GC cells. Data are presented as mean ± SD for at least three independent experiments. (**E**) Wound-healing assays revealed that both GSK2256098 and si*-FAK* could quantitatively reduce the *CPNE8*-induced migration in BGC823 cells using Image J software. (**F**) Transwell assays confirmed that GSK2256098 or knocking down *FAK* could inhibit migration and invasiveness of GC cells with overexpression of *CPNE8*. (**G**) The representative images showed the adhesion ability of GC cells with knockdown or overexpression of *CPNE8* compared to the control cells and the attenuation of BGC823-oe-*CPNE8* with GSK2256098 or knockdown of* FAK*. The number of GC cells shown in the bar graph that adhered to the plates coated after 2h of incubation was quantified using Image J software.

We investigated the potential mechanism of action of *CPNE8* in GC metastasis. GSEA was performed based on the median expression levels of *CPNE8*, Gene Set Enrichment Analysis (GSEA) was performed in the TCGA-STAD cohort. A set of signaling pathways, such as focal adhesion, gap junction, and ECM receptor interaction, was functionally enriched in GC patients with high *CPNE8* expression (Supplementary Figure S3A). In addition, we screened genes with strong correlations with *CPNE8* expression (|R|≥0.4 and p≤0.05) using the Cbioportal website for KEGG functional enrichment analysis in the TCGA-STAD cohort. We found that the focal adhesion pathway, cell adhesion molecule (CAMs) pathway, and tight junctions were abundant (Supplementary Figure S3B). These signaling pathways are closely associated with tumor metastasis 35-37. We performed mass spectrometry analysis on *CPNE8* knockdown cells to screen for differentially expressed proteins for KEGG functional enrichment. Pathway analysis indicated that the genes regulating the focal adhesion pathway were most significantly disrupted after *CPNE8* knockdown (enrichment score =2.5, p <0.01, **Figure 4A**).

Focal adhesion has been recognized as an essential step in cancer cell migration and invasion, and can activate several signaling pathways through phosphorylation and protein-protein interactions that promote tumorigenesis and metastasis 35, 38. To explore whether *CPNE8*-mediated metastasis of GC was regulated through focal adhesion, we examined the expression of *FAK* and the target gene ERK at the mRNA and protein levels by qRT-PCR and western blotting, respectively. Our results revealed that knockdown of *CPNE8* in AGS cells markedly decreased *FAK*, p-*FAK*, total Erk1/2, and p-ERK expression at both the mRNA and protein levels. By contrast, BGC823 cells overexpressing *CPNE8* exhibited significantly increased *FAK* and p-*FAK* at both the mRNA and protein levels, and increased p-ERK mRNA and protein levels, but with no significant change in total Erk1/2 (**Figure 4B-C**). To determine the effect of the focal adhesion pathway on gastric carcinogenesis and metastasis, we used siRNA and the *FAK* inhibitor GSK2256098 to treat *CPNE8* overexpressed BGC823 cell lines to detect cell proliferation, motility, and invasiveness, respectively. We treated BGC823-oe-*CPNE8* cells with different concentrations (0-10 μM) of GSK2256098 and incubated them at 37 °C for 2 h before detecting the expression levels of *FAK* by Western blotting. We found that treatment with GSK2256098 at a concentration of 10 μM significantly reduced *FAK* levels (Supplementary Figure S3C). Therefore, this concentration was selected for subsequent inhibitor assays. We also examined *FAK* levels by Western blotting to verify the knockdown efficiency of si-*FAK* (Supplementary Figure S3D).

We performed an EDU staining experiment to demonstrate the effect of attenuated *FAK* expression on proliferative capability. As shown in **Figure 4D**, the result illustrated that the growth capacity of BGC823-oe-*CPNE8* cells decreased after knockdown of *FAK* or GSK2256098 treatment. In addition, we conducted cell cycle and apoptosis experiments to demonstrate the influence of tumor carcinogenesis with after weakening *FAK* expression. As shown in Supplementary Figure S4A, elevated *CPNE8* had no effect on the cell cycle with GSK2256098 treatment or knockdown of *FAK*. After knockdown of *FAK* expression, there was a slight increase apoptotic cells in BGC823-oe-*CPNE8* cells, while no apoptosis was induced by inhibitor treatment (Supplementary Figure S4B). These results suggested that *CPNE8* might participate in other pathways to regulate cell growth.

The wound-healing assay showed that the mobility of BGC823-oe-*CPNE8* cells was diminished in cells treated with si-*FAK* and GSK2256098 (**Figure 4E**). Furthermore, the Transwell assay showed that GSK2256098 reversed the *CPNE8*-induced migration and invasion abilities of GC cells (**Figure 4F**), and similar findings were observed after knockdown of *FAK* with si-*FAK*. These results indicate that the focal adhesion pathway mediates *CPNE8*-induced cell migration and invasion, which can be entirely reversed by the *FAK* inhibitor GSK2256098 or knockdown of *FAK*. In addition, we tested the effect of *CPNE8* on cell adhesion by using an adhesion assay. As shown in **Figure 4G**, knockdown of *CPNE8* in AGS cells resulted in significantly fewer adherent cells than in control cells. In contrast, the overexpression of *CPNE8* in BGC823 cells enhanced their ability to adhere to the stroma. Consistently, treatment with GSK2256098 or siRNA targeting *FAK* resulted in significant reductions in the number of adherent BGC823-oe-*CPNE8* cells. These results strongly supported our conclusion that *CPNE8* promotes GC metastasis via upregulation of the focal adhesion pathway.

### CPNE8 overexpression enhances GC metastasis *in vivo*

**Figure 5:**
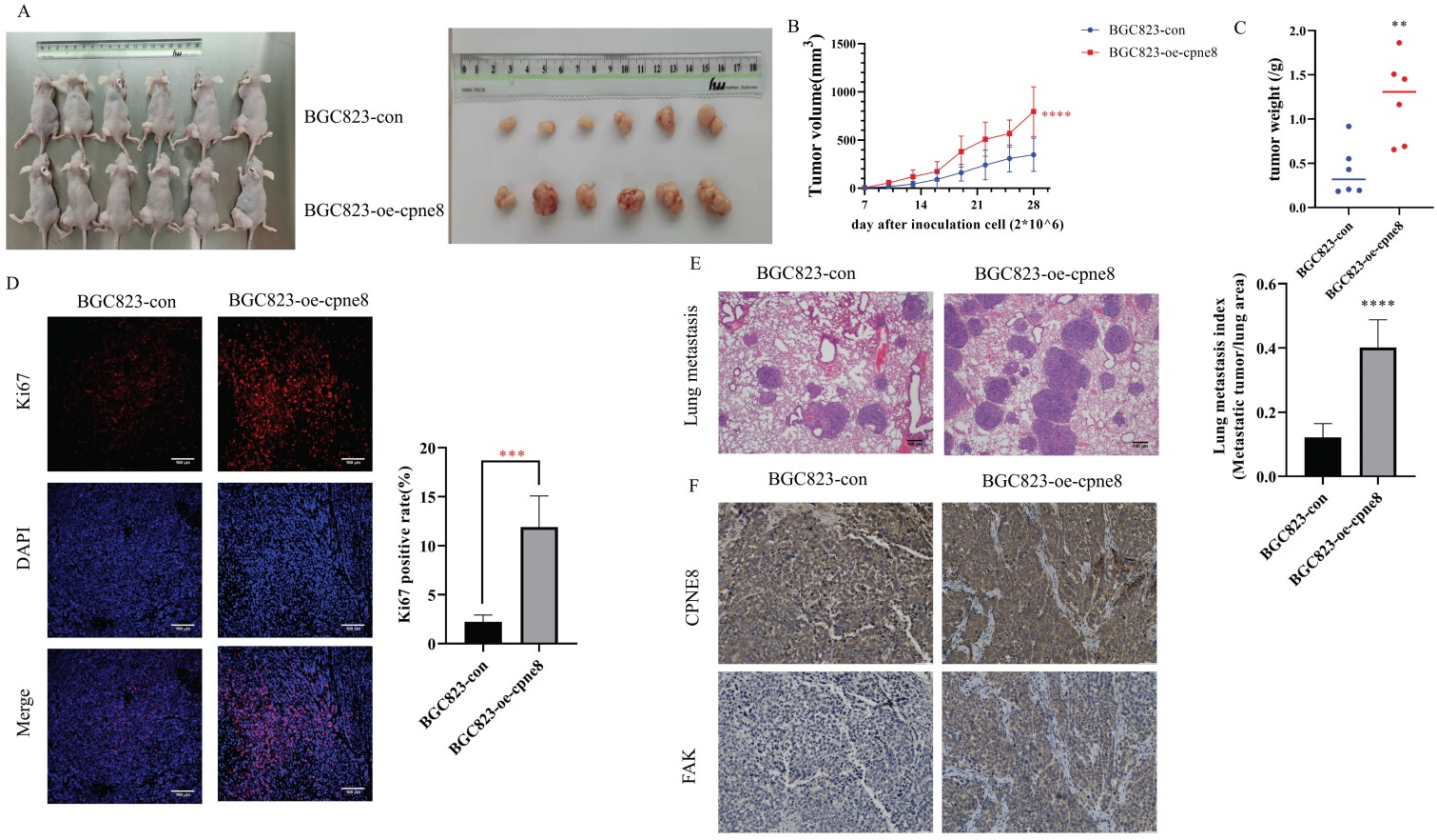
*CPNE8* overexpression enhanced GC metastasis *in vivo*. (**A**) The representative image demonstrated the tumor-bearing mice subcutaneously injected with BGC823 cells overexpressing *CPNE8* versus control mice and the tumors harvested from the nude mice. (**B**) Tumor volumes were recorded every 3 days. Data are represented as mean ± SD of five mice in each group. (**C**) Significant increase in tumor weight of mice with overexpression of *CPNE8*. (**D**) Immunofluorescence staining of the proliferation marker revealed a higher expression of Ki67 in tumors overexpressing *CPNE8*. (**E**) Representative images of HE staining of lungs were collected from mice injected with GC cells *via* tail vein. Results are also expressed as metastatic lung index (lung tumor area/total lung area) analyzed in mice bearing the metastasis. (**F**) Immunohistochemistry staining of *CPNE8* and *FAK* in GC tissues harvested from the xenograft mice.

The effect of *CPNE8* on GC metastasis was investigated *in vivo*. Mice injected with BGC-823-oe-*CPNE8* cells showed a significant increase in tumor size and growth at 4 weeks post-inoculation compared to those injected with control cells (**Figure 5A-B**). After sacrificing the mice, tumor tissues were weighed. The group with *CPNE8* overexpression showed a significant increase in tumor weight (**Figure 5C**). Immunofluorescence staining confirmed that tumors derived from the BGC823-oe-*CPNE8* group exhibited higher Ki67 expression levels than tumors derived from control cells (**Figure 5D**). This implies that *CPNE8* could improve the proliferative capacity of tumors *in vivo*. To further define the role of *CPNE8* expression in promoting metastasis *in vivo*, we injected BGC823 cells with *CPNE8* overexpression into nude mice through the tail vein. All mice were sacrificed four weeks later to harvest the lungs for H&E staining. Nude mice inoculated with *CPNE8* overexpressing BGC823 cells exhibited more metastatic lung nodules (**Figure 5E**). Immunohistochemical analysis showed a consistent trend of *CPNE8* and *FAK* expression in GC tissues of xenograft mice. IHC staining confirmed that tumors derived from the BGC823-oe-*CPNE8* group exhibited higher *CPNE8* and *FAK* expression levels than tumors derived from control cells (**Figure 5F**). Taken together, these *in vivo* experiments verified that *CPNE8* was essential for promoting the growth and metastasis of GC cells.

### Influence of high CPNE8 expression on the infiltration of CAFs in GC

**Figure 6:**
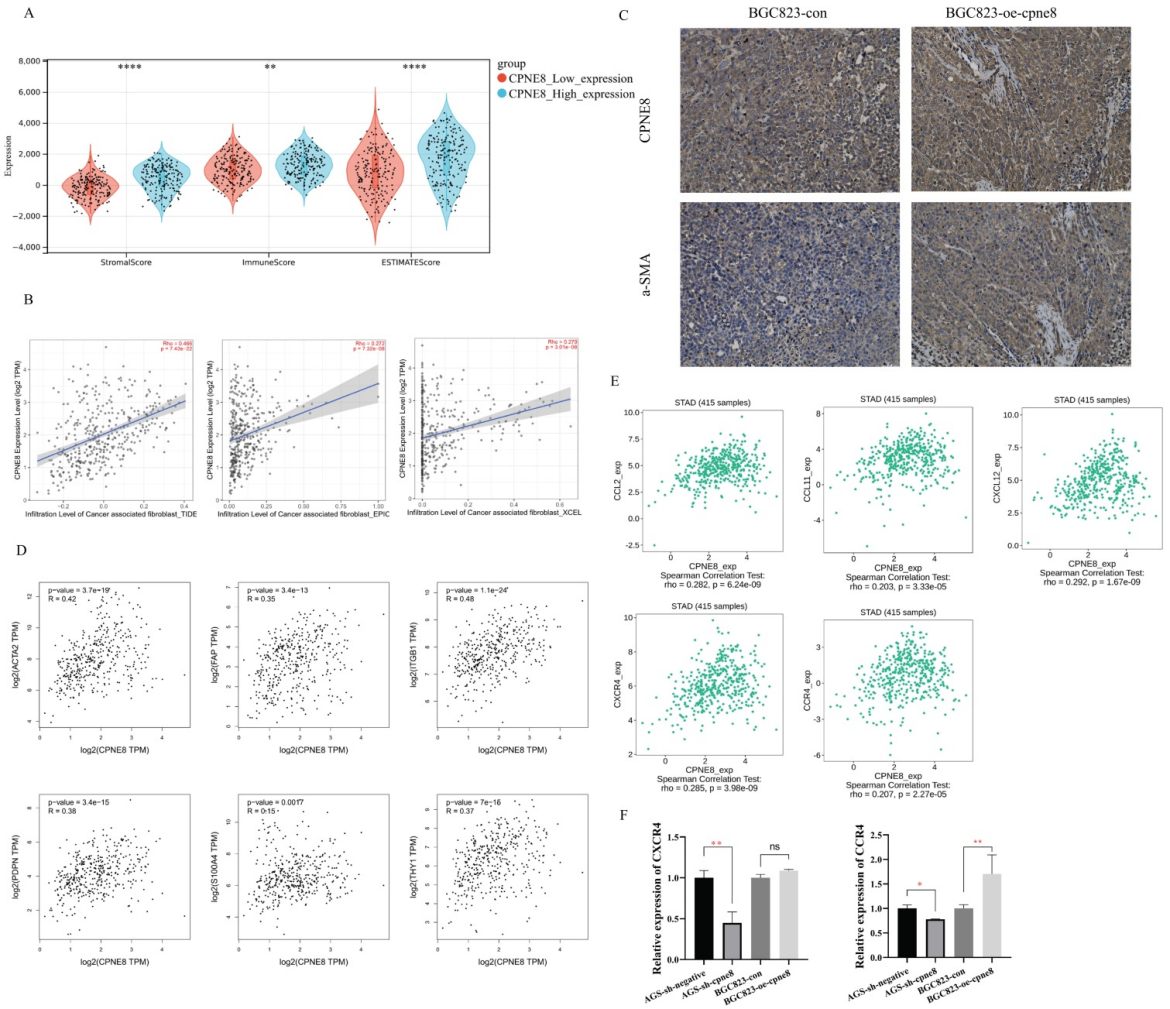
High expression of *CPNE8* was associated with the infiltration of cancer-associated fibroblasts in GC. (**A**) Manipulated Stromal score, Immune score, and ESTIMATE score based on *CPNE8* status in TCGA-STAD cohort. (**B**) The correlation of *CPNE8* expression and the infiltration of CAFs was quantified using the TIDE, EPIC, and XCELL algorithm in the TCGA-STAD cohort in the TIMER2 database. Each dot represents one sample. (**C**) Immunohistochemistry staining of *CPNE8* and a-SMA in GC tissues of xenograft mice. (**D**) Significant and positive correlations between *CPNE8* expression and *ACTA2*, *FAP*, *ITGB1*, *PDPN*, *S100A4*, and *THY1*. (**E**) The correlation of *CPNE8* expression with various chemokines or chemokine receptors was displayed using the TISIDB database. (**F**) The mRNA levels of chemokine receptors were quantified by qRT-PCR in GC cells with respective knockdown or overexpression of *CPNE8* compared to the control cells.

Interactions between tumor cells and tumor microenvironment (TME) dynamically regulate the metastatic process 39-41. We reanalyzed the TCGA-STAD dataset using the ESTIMATE algorithm and calculated the stromal score, immune score, and tumor purity (**Figure 6A**). The results showed that the stromal score (p <0.001), immune score (p <0.01), and ESTIMATE score (p <0.001) were significantly higher in the high *CPNE8* expression cluster (**Figure 6A**). Consistently, we found that high *CPNE8* expression was associated with higher stromal, immune, and ESTIMATE scores compared to the low *CPNE8* group in GSE84437 (Supplementary Figure S5A).

Cancer-associated fibroblasts (CAFs), the most prominent and abundant cell type in the GC stroma, are activated fibroblasts that play a key role in GC progression and metastasis 42-44. We used multiple algorithms to compare the association between CAFs infiltration and *CPNE8* expression. Consistent results demonstrated a high correlation between *CPNE8* and CAF infiltration in the TCGA-STAD cohort (**Figure 6B**) and GSE84437 (Supplementary Figure S5B). These results suggest that *CPNE8* may manipulate the recruitment of CAFs into the tumor microenvironment. IHC staining verified that tissues in xenograft mice overexpressing *CPNE8* exhibited high expression of α-SMA, a well-known CAF marker (**Figure 6C**). In addition, we performed the correlation analysis between *CPNE8* expression and several activated CAFs biomarkers in the TCGA-STAD cohort, such as α-smooth muscle actin (α-SMA, *ACTA2*), fibroblast-specific protein-1 (FSP-1, *S100A4*), fibroblasts activated protein (*FAP*), Thy-1 (*THY1*), (*PDPN*), and integrin beta1 (*ITGB1*). As shown in **Figure 6D**, there were significant positive correlations between *CPNE8* and activated CAFs biomarkers, such as *ACTA2* (R =0.42, p =3.7e-19), *FAP* (R =0.35, p =3.4e-13), *ITGB1* (R =0.48, p =1.1e-24), *PDPN* (R =0.38, p =3.4e-15), *S100A4* (R =0.15, p =0.0017), and *THY1* (R =0.37, p =7e-15). CAFs always secret cytokines, chemokines, and growth factors in the tumor microenvironment to promote tumor progression and migration 45-47. To test this in our model, we analyzed the correlation between CPEN8 and various chemokines and chemokine receptors using the TISIDB database. As shown in **Figure 6E**, there were significant positive correlations between *CPNE8* and chemokines secreted from CAFs, such as *CCL2* (R =0.282, p =6.24e-09), *CCL11* (R =0.203, p =3.33e-05), and *CXCL12* (R =0.292, p = 1.67e-09). We also observed a correlation between *CPNE8* expression and chemokine receptors, including *CXCR4* (R =0.285, p =3.98e-09) and *CCR4* (R =0.207, p =2.27e-05). We used qRT-PCR analysis to verify the correlation between *CPNE8* and chemokine receptor binding to the chemokines secreted by CAFs. The results showed that knockdown of *CPNE8* resulted in a significant decrease in the expression of *CXCR4* and *CCR4* in GC cells compared to control cells, while overexpression of *CPNE8* resulted in an upregulation of these genes (**Figure 6F**). These findings suggested that high *CPNE8* expression may affect the infiltration of cancer-associated fibroblasts.

### Higher expression of CPNE8 was correlated with poorer immunotherapy efficacy

**Figure 7:**
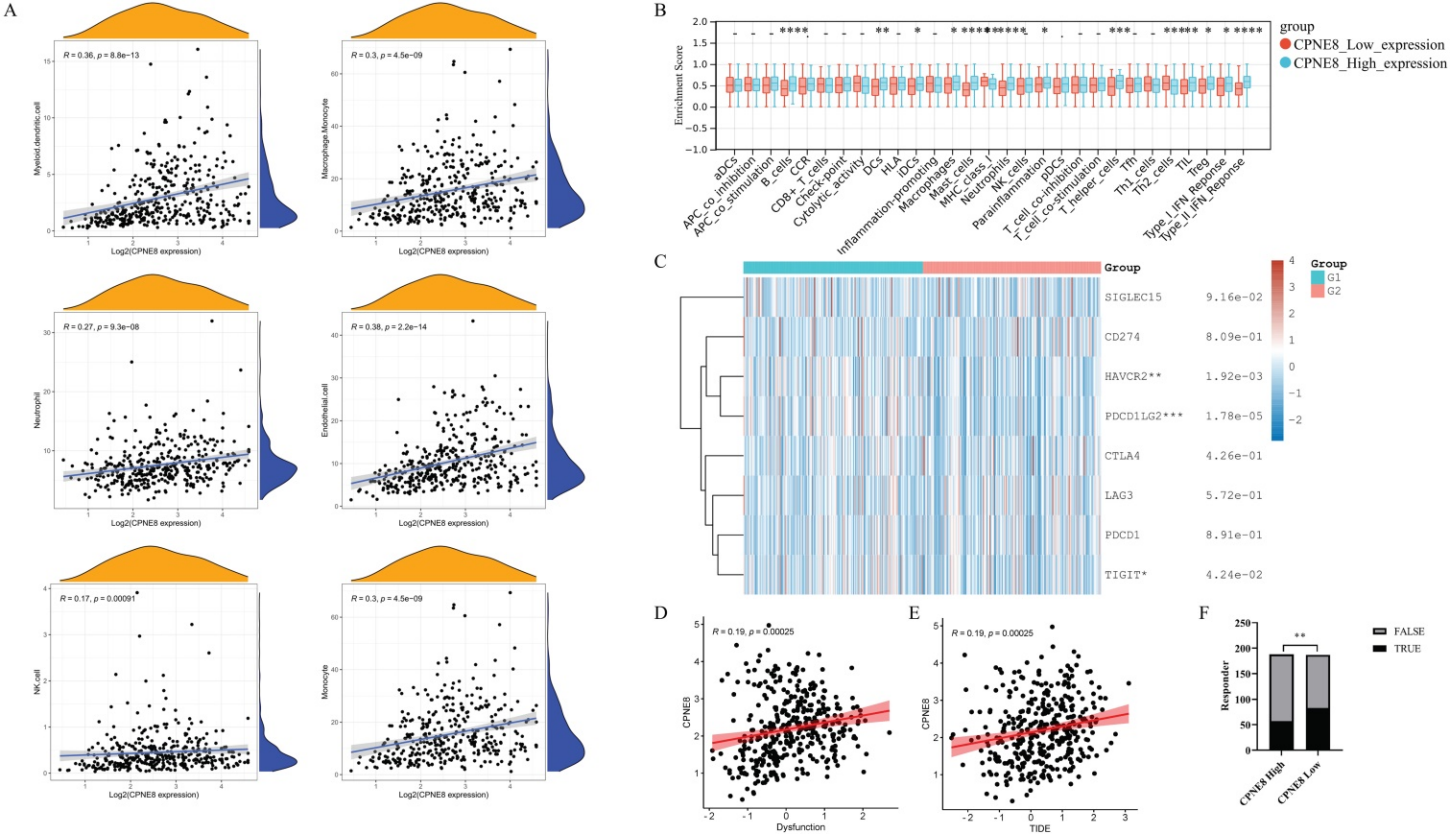
*CPNE8* expression could predict the clinical benefit of ICB. (**A**) Correlation of *CPNE8* expression and immune infiltrating cells quantified using the MCP-counter algorithm in the TCGA-STAD cohort. (**B**) Correlation between ssGSEA scores of 29 immune cells and *CPNE8* expression in gastric cancer. (**C**) The heatmap showed the correlation between *CPNE8* expression and immune checkpoint genes. G1 represented the *CPNE8* high expression group; G2 represented the *CPNE8* low expression group. (**D**) Increased *CPNE8* expression was associated with higher immune dysfunction scores. (**E**) The mRNA expression of *CPNE8* was positively correlated with the TIDE score. (**F**) The rate of immune checkpoint blockade responses in GC patients was predicted by the TIDE algorithm in the high or low *CPNE8* groups.

Immune cells in the tumor microenvironment (TME) play an essential role in tumor progression 48. We used the MCP-counter algorithm to analyze the relationship between *CPNE8* expression and immune cell infiltration and found high correlations between *CPNE8* expression and monocyte, macrophage/monocyte, myeloid dendritic cell, and neutrophil cell infiltration (**Figure 7A**). In addition, we used the “ssGSEA” algorithm to calculate the absolute enrichment of immune pathways in each sample and found that signaling pathways involved in immune responses, such as B cells, DCs, macrophages, MHC class I, Type I IFN Reponse, and Type II IFN Reponse, were enriched in the high *CPNE8* expression cluster (**Figure 7B**). To further elucidate the role of *CPNE8* in immunotherapy, as represented by immune checkpoint blockades CB), we extended our analysis to the association of *CPNE8* with several well-known biomarkers. Spearman's correlation analysis of the TCGA-STAD cohort revealed that *CPNE8* expression was significantly negatively correlated with MSI (R = -0.25, p = 8.87e-07) and TMB (R =-0.36, p =9.62e-13), implying a poor response to immunotherapy (Supplementary Figure S6A-B). In the TCGA-STAD cohort, *CPNE8* expression was positively correlated with the expression of immune checkpoint genes such as *PDCD1LG2*,* HAVCR2*, and* TIGIT*, suggesting that higher *CPNE8* expression may predict poorer immune responses in GC patients (**Figure 7C**). Furthermore, using the TIDE database (http://tide.dfci.harvard.edu), we calculated the correlations of *CPNE8* with dysfunction and TIDE scores and found that increased *CPNE8* expression was associated with higher immune dysfunction (R =0.19, p =0.00025) (**Figure 7D**) and TIDE scores (R =0.19, p =0.00025) (**Figure 7E**). Consistently, high *CPNE8* expression predicted adverse responses to immune checkpoint therapy in GC patients (efficiency 30.32% vs. 44.39%, p =0.0049) (**Figure 7F**). These results suggest that *CPNE8* expression can predict the clinical benefits of ICB in STAD.

## Discussion

Tumor metastasis, the leading cause of death in patients with advanced GC, remains a poorly understood cause of tumor progression 6, 9. Therapeutic efficacy for metastatic GC remains limited owing to the poor prognosis of patients 49. Accumulating evidence suggests that CPNE family members are involved in tumorigenesis and metastasis. However, the role of *CPNE8* in GC progression and metastasis has not yet been thoroughly evaluated. Furthermore, tumor metastasis is not only driven by the accumulation of intrinsic changes within malignant cells, but is also modulated by various immune and stromal components in the tumor microenvironment 50-52. Therefore, the aim of this study was to explore the potential mechanisms of *CPNE8* in the progression and metastasis of GC and its potential immune activation and sensitivity to immunotherapies of GC patients.

Our research revealed that *CPNE8* is highly expressed in GC, with significantly higher expression in metastatic tumors, especially in patients with liver metastases. In addition, *CPNE8* was highly expressed in patients with advanced tumors (with a progressive grade or advanced TNM stage), suggesting that patients with high *CPNE8* expression exhibit more aggressive clinicopathological characteristics. *CPNE8* could be used as an independent prognostic factor for GC, and higher *CPNE8* expression predicted poorer prognosis in GC. Summarily, *CPNE8* may serve as a novel prognostic marker for GC. We knocked down or overexpressed *CPNE8* in GC cells. *In vitro* and *in vivo* functional assays revealed that knockdown of *CPNE8* reduced cell proliferation, possibly by promoting apoptosis and inhibiting the G1/S phase of cells. In contrast, overexpression of *CPNE8* promoted cell proliferation by facilitating transition from the G1/S phase. Moreover, our results showed that GC cells with high *CPNE8* expression demonstrated enhanced invasion and migration abilities, thus providing a basis for tumor metastasis. *In vivo*, *CPNE8* promoted the growth and metastasis of xenograft tumors, which was consistent with the results obtained *in vitro*.

We further analyzed the mechanism of *CPNE8* in enhancing GC metastasis. First, the GSEA and KEGG results showed that the high *CPNE8* expression group was functionally enriched in focal adhesion, gap junction, and ECM receptor interaction in the TCGA-STAD cohort, which were correlated with cancer metastasis 35-37. Our mass spectrometry analysis confirmed that *CPNE8* is closely associated with focal adhesions. The role of focal adhesion in cell adhesion, migration, and cancer invasiveness has been well-studied 35, 53, 54. The tyr397 phosphorylation and kinase activity of *FAK* were critical for GC invasiveness 55. *ERK* is a member of the mitogen-activated protein kinase (*MAPK*) family and is activated by *FAK* interactions 56, 57. Our study demonstrated that *CPNE8* enhanced GC cell migration and invasion in an *FAK*-dependent manner. The phosphorylation levels of *FAK* (Tyr397) and *ERK* (p42/p44) decreased in cells with *CPNE8* knockdown and increased in cells with *CPNE8* overexpression. GSK2256098, a small molecule *FAK* inhibitor, reversed *CPNE8*-induced GC cell proliferative capability, mobility, and invasiveness. The proliferative capability of GC cells decreased after GSK2256098 treatment or knocking down *FAK*. However, there were no significant differences in the cell cycle and apoptosis between BGC823-oe-*CPNE8* cells with and without the *FAK* inhibitor. Based on the findings from cell cycle and apoptosis analyses, knockdown of *CPNE8* led to G1-S phase delay and apoptosis, while overexpression of *CPNE8* promoted the cell cycle, suggesting that *CPNE8* may regulate the G1-S phase transition and cell apoptosis via alternative pathways to focal adhesion.

We further studied tumor cell adhesion using fibronectin, an essential component for the adhesion of many cell types 58. While our results showed that *CPNE8* promoted GC cell adhesion, which could be reduced by *FAK* inhibitor, the underlying molecular mechanism between *CPNE8* and *FAK* is still unknown. Nevertheless, our results suggest that *CPNE8* plays a carcinogenic role in GC by activating the focal adhesion pathway, and *FAK* inhibition may be a promising therapy for GC patients with high *CPNE8* expression.

Several studies have reported that the tumor immune microenvironment, consisting of immune cells, inflammatory cells, and CAFs, is associated with the invasion and metastasis of multiple tumor cells, and is closely correlated with tumor prognosis 50, 51. CAFs, critical components of the stroma, have been reported to regulate chemokines in the tumor microenvironment and promote the progression of tumor metastasis 45, 46. Our results demonstrated that *CPNE8* was positively correlated with CAFs using different algorithms in TCGA-STAD and GSE84437 cohorts. IHC analysis results also revealed that overexpression of *CPNE8* in GC tissue exhibited higher α-SMA expression, an evidential marker of CAFs, which implied that *CPNE8* overexpression might recruit more CAFs in the microenvironment to promote GC metastasis. It has been previously demonstrated that CAFs secrete *CXCL12*, thus stimulating *CXCL12*/*CXCR4* signaling and promoting tumor growth and angiogenesis in GC 59. Previous studies also showed that CAFs could produce the chemokine ligand *CCL17* to act on *CCR4* of cancer cells to improve cancer proliferation and migration 60. In addition, Tsuyada et al. showed that *CCL2* and* CXCL14* secreted by CAFs can increase the recruitment of macrophages and promote the invasive ability of breast cancer 61. Nieh et al. found that CAFs facilitate cancer invasiveness through paracrine effects on micro-environmental *CCL11* signaling in oral squamous cell carcinoma 62. Our study revealed significant correlations between CPNR8 expression and chemokines secreted from CAFs in the TCGA-STAD cohort using the TISIDB database.

Interestingly, we assessed the mRNA levels of the chemokine receptors *CCR4* and *CXCR4* in GC cells and found that knockdown or overexpression of *CPNE8* significantly regulated their expression, implying that *CPNE8* may affect the function of CAFs via modulation of chemokine binding to cancer cells. However, whether mutual regulation between *CPNE8* and CAFs exists is still unclear. The potential regulatory mechanism between *CPNE8* and CAFs warrants more in-depth study, which may contribute to the understanding of additional mechanisms involved in GC metastasis.

Multiple tumor immune infiltrating cells, such as macrophages, MDSC, granulocytes, and lymphocytes, have been reported to promote multiple tumor metastases 39, 63. In this study, we showed that *CPNE8* expression is significantly positively correlated with monocytes, macrophages/monocytes, myeloid dendritic cells, and neutrophils. Functional enrichment by “ssGSEA” also demonstrated the involvement of *CPNE8* in immune-related pathways.

Tumor immunotherapy is a powerful and promising clinical approach for the treatment of patients with cancer. However, the response rate remains challenging to assess in tumors, including in patients with GC 64, 65. An increasing number of biomarkers are used to predict immune response, and these biomarkers play vital roles in the development, metastasis, and treatment of cancer 66, 67. Microsatellite instability (MSI) is the first pan-cancer biomarker approved for immune checkpoint blockade (ICB) therapy 68, 69. MSI-induced shift mutations result in the generation of large neoantigens, and tumors with high mutation rates may respond well to checkpoint inhibitors (CPIs) 70. TMB has been reported as an effective biomarker of immune checkpoint inhibitor sensitivity 71. Tumors with high TMB typically have higher levels of immune system-recognizable neoantigens that correlate with the response to PD-1, PD-L1, and CTLA-4 blockade immunotherapy 71, 72. In this study, we observed a significant negative correlation between *CPNE8* expression and MSI/TMB, implying a poor immune response in GC patients with high *CPNE8* expression. Although the immune system can recognize malignant cells, the inactivation of anti-tumor T cells due to the upregulation of suppressive immune checkpoints in the tumor microenvironment results in an ineffective immune system response to the tumor cells 73. We found that *CPNE8* expression was positively correlated with the expression of suppressive checkpoints—*HAVCR2*, *PDCD1LG2*, and *TIGIT*. Peng et al. designed a new computational framework, the TIDE score, to assess the status of tumor immunity and predict the effect of immunosuppressive therapy 28, 29. *CPNE8* expression was positively correlated with immune dysfunction and TIDE scores in our study. By contrast, the response rate to immune checkpoint therapy was reduced by 14.07% in the *CPNE8* high-expression group. These results suggest that GC patients with high *CPNE8* expression may benefit less from available immune blockade therapy.

Our findings establish a novel link between *CPNE8* expression and GC progression. *CPNE8* might promote tumor proliferative capacity by accelerating the G1-S phase transition of the cell cycle. Conversely, knockdown of *CPNE8* could lead to G1-S phase blockade to induce apoptosis, an outcome of cell cycle arrest 74-76. In addition, *CPNE8*, a critical core factor of tumor metastasis, triggers the focal adhesion pathway by upregulating *FAK* and *ERK* expression and activating CAFs in the tumor microenvironment by regulating chemokines and their receptors, leading to a suppressed immune response.

Our study had some limitations, we did not elucidate the molecular mechanism of *FAK* regulation by *CPNE8* and how *CPNE8* activates CAFs. While studies concerning *FAK* activation in CAFs have been reported 77, 78, it is worthwhile to further investigate whether *CPNE8* is involved in this regulatory effect.

In conclusion, our findings suggest that *CPNE8* represents an independent prognostic risk factor that can predict poor outcomes in GC. *CPNE8* could affect the migration and invasion of GC cells by enhancing focal adhesion, thus identifying a promising therapeutic target for GC. Furthermore, high *levels of CPNE8* can recruit more CAFs and involve immune pathways in the tumor microenvironment. Together, these findings may help develop more effective therapeutic strategies by targeting GC progression and metastasis.

## Supplementary Material

Supplementary figures and tables.

## Figures and Tables

**Table 1:** Relationship between CPNE8 expression and clinicopathologic Characteristics of TCGA-STAD patients (n=322)

Characteristics	CPNE8 expression		χ2	P-value
	Low (n=161)	High (n=161)		
Gender				
Female	61	60	0.01324	0.9084
Male	100	101		
Grade				
G1 + G2	70	50	5.314	0.0212^*^
G3	91	111		
Invasion depth				
T1 + T2	41	33	1.123	0.2893
T3 + T4	120	128		
Lymph metastasis				
N0 + N1	93	76	3.599	0.0578
N2 + N3	68	85		
Distant metastasis				
M0	154	146	3.122	0.0772
M1	7	15		
TNM stage				
Stage I + II	86	69	3.595	0.058
Stage III + IV	75	92		
*p <0.05				

## Abbreviations

AUC: area under curve
CAFs: cancer associated fibroblasts
CAMs: cell adhesion molecules
CPI: checkpoint inhibitor
CPN: copine
DFS: disease-free survival
FAK: focal adhesion kinase
FAP: fibroblast-activated protein
FBS: fetal bovine serum
FSP: fibroblast-specific protein
GC: gastric cancer
GES1-: gastric mucosal epithelial cell line
GO: gene ontology
GSEA: gene-set enrichment analysis
ICB: immune checkpoint blockade
ITGB1: integrin beta 1
KEGG: Kyoto encyclopedia of genes and genomes
MAPK: mitogen-activated protein kinase
MSI: microsatellite instability
OS: overall survival
PDPN: podoplanin
PFS: progression-free survival
ROC: receiver operating characteristic
ssGSEA: single-sample gene set enrichment analysis
STAD: clinical data of stomach adenocarcinoma
TIDE: tumor immune dysfunction and exclusion
TME: tumor microenvironment
