# Supplementary material for: CPNE8 Promotes Gastric Cancer Metastasis by Modulating Focal Adhesion Pathway and Tumor Microenvironment
Source: Int J Biol Sci. 2022 Jul 25;18(13):4932–49. doi: 10.7150/ijbs.76425 (PMC9379401; doi:10.7150/ijbs.76425)
Supplement: Supplementary file 1 — Supplementary figures and tables. [file ijbsv18p4932s1.pdf]

**SUPPLEMENTARY MATERIAL**

**Supplementary Figure S1** (A) The expression of *CPNE8* at different metastatic areas was compared in the TCGA-STAD cohort. (B) *CPNE8* showed poorer performance in diagnosing gastric cancer than CEACAM5 in the TCGA-STAD cohort. (C) *CPNE8* exhibited excellent performance in diagnosing gastric cancer than CEACAM5 in GSE118946 dataset. (D)The expression of *CPNE8* showed no difference between male and female GC patients. (E) There was no difference in *CPNE8* expression among various T classifications. (F) The expression of *CPNE8* did not differ among the four N classifications.

**Supplementary Figure S2** (A) Univariate Cox regression analyses estimating prognostic value (OS) of *CPNE8* in TCGA-STAD cohort. HR >1.0 indicates overexpression *CPNE8* is an unfavorable prognostic biomarker. (B) Multivariate Cox regression analysis for estimating the predictive value (OS) of *CPNE8* in the TCGA-STAD cohort. HR >1.0, $p < 0.05$ indicates that *CPNE8* can serve as an independent prognostic risk factor biomarker. Receiver operating characteristic (ROC) curves for risk scores predicting overall survival (C), progression-free survival (D), and disease-free survival (E) at 1/3/5 years in the TCGA-STAD cohort.

**Supplementary Figure S3** (A) The plot showed enrichment of metastasis-related signaling pathways in the high *CPNE8* expression group by GSEA analysis in the TCGA-STAD cohort. (B) KEGG functional enrichment analysis revealed a significant abundance of *CPNE8* correlated genes in metastasis-related signaling, such as focal adhesion, tight junction, and cell adhesion molecules. (C) The results showed the protein level of *FAK* after adding the small molecule *FAK* inhibitor GSK2256098 to treat for 2 hours. Relative protein expression (interested protein/*GAPDH*) was quantified in GC cells. The optimal concentration was selected as 10 μM. (D) The results showed the knockdown efficiency of si-*FAK* by detecting *FAK* protein level.

Relative protein expression was also quantified by Image J software and *GAPDH* expression was used as a normalized control.

**Supplementary Figure S4** (A) Cell cycle analysis of BGC823-oe-*CPNE8* cells after attenuating *FAK* by GSK2256098 treatment or knocking down *FAK*. The bar graph represented the proportion of cells in various cell cycle phases in GC cells. Data are presented as mean ± SD for at least three independent experiments. (B) Analysis of apoptosis in GC cells after GSK2256098 treatment or knockdown of *FAK*. The proportion of apoptotic cells in GC cells was shown as column graphs. Data are presented as mean ± SD for at least three independent experiments.

**Supplementary Figure S5** (A) Different expression of ESTIMATE score, Immune score, and Stromal score was calculated based on *CPNE8* status in GSE84437 cohort. (B) Correlation of *CPNE8* expression and the infiltrating of CAFs quantified using the MCP-counter algorithm in the GSE84437 cohort.

**Supplementary Figure S6** (A) Spearman correlation analysis of MSI scores and *CPNE8* gene expression was negatively correlated. (B) Spearman correlation analysis of TMB sores and *CPNE8* gene expression was negatively correlated.

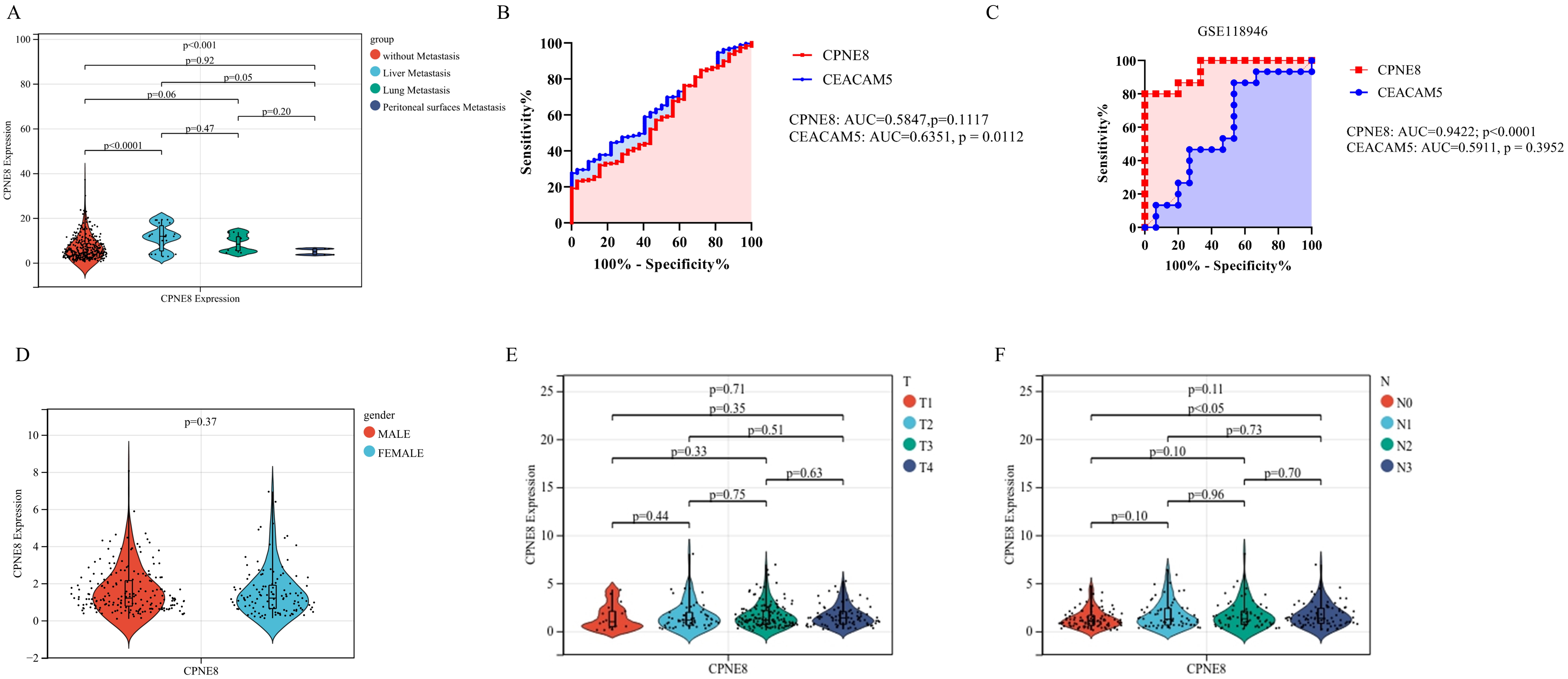

A
group
without Metastasis
Liver Metastasis
Lung Metastasis
Peritoneal surfaces Metastasis
p<0.001
p=0.92
p=0.05
p=0.06
p=0.20
p=0.47
p<0.0001
CPNE8 Expression
CPNE8 Expression
B
CPNE8
CEACAM5
CPNE8: AUC=0.5847,p=0.1117
CEACAM5: AUC=0.6351, p = 0.0112
Sensitivity%
100% - Specificity%
C
GSE118946
CPNE8
CEACAM5
CPNE8: AUC=0.9422; p<0.0001
CEACAM5: AUC=0.5911, p = 0.3952
Sensitivity%
100% - Specificity%
D
gender
MALE
FEMALE
p=0.37
CPNE8 Expression
CPNE8
E
T
T1
T2
T3
T4
p=0.71
p=0.35
p=0.51
p=0.33
p=0.63
p=0.75
p=0.44
CPNE8 Expression
CPNE8
F
N
N0
N1
N2
N3
p=0.11
p<0.05
p=0.73
p=0.10
p=0.70
p=0.96
p=0.10
CPNE8 Expression
CPNE8

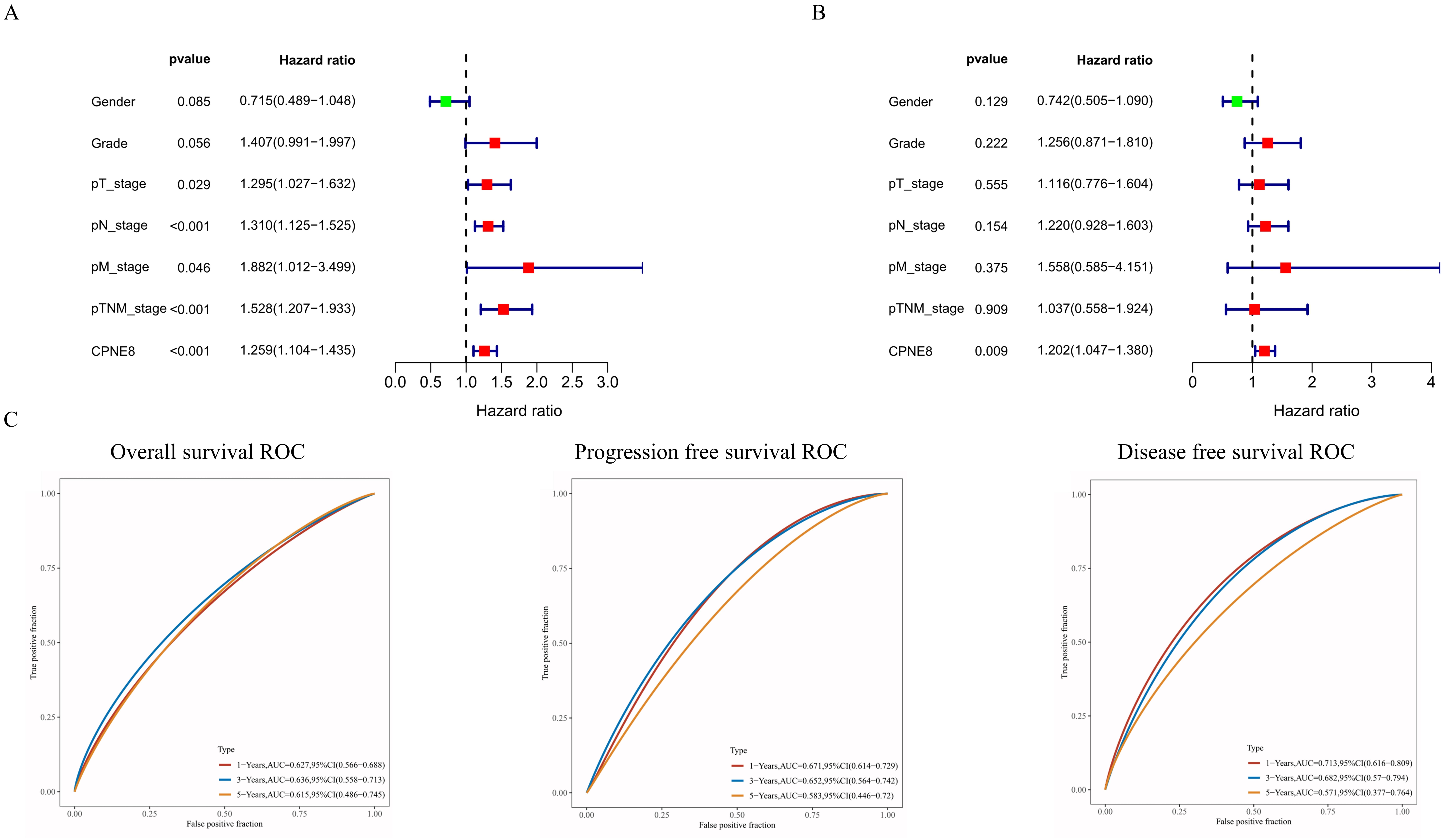

A
pvalue
Hazard ratio
Gender 0.085 0.715(0.489−1.048)
Grade 0.056 1.407(0.991−1.997)
pT_stage 0.029 1.295(1.027−1.632)
pN_stage <0.001 1.310(1.125−1.525)
pM_stage 0.046 1.882(1.012−3.499)
pTNM_stage <0.001 1.528(1.207−1.933)
CPNE8 <0.001 1.259(1.104−1.435)
0.0 0.5 1.0 1.5 2.0 2.5 3.0
Hazard ratio
B
pvalue
Hazard ratio
Gender 0.129 0.742(0.505−1.090)
Grade 0.222 1.256(0.871−1.810)
pT_stage 0.555 1.116(0.776−1.604)
pN_stage 0.154 1.220(0.928−1.603)
pM_stage 0.375 1.558(0.585−4.151)
pTNM_stage 0.909 1.037(0.558−1.924)
CPNE8 0.009 1.202(1.047−1.380)
0 1 2 3 4
Hazard ratio
C
Overall survival ROC
True positive fraction
False positive fraction
Type
1−Years,AUC=0.627,95%CI(0.566−0.688)
3−Years,AUC=0.636,95%CI(0.558−0.713)
5−Years,AUC=0.615,95%CI(0.486−0.745)
Progression free survival ROC
True positive fraction
False positive fraction
Type
1−Years,AUC=0.671,95%CI(0.614−0.729)
3−Years,AUC=0.652,95%CI(0.564−0.742)
5−Years,AUC=0.583,95%CI(0.446−0.72)
Disease free survival ROC
True positive fraction
False positive fraction
Type
1−Years,AUC=0.713,95%CI(0.616−0.809)
3−Years,AUC=0.682,95%CI(0.57−0.794)
5−Years,AUC=0.571,95%CI(0.377−0.764)

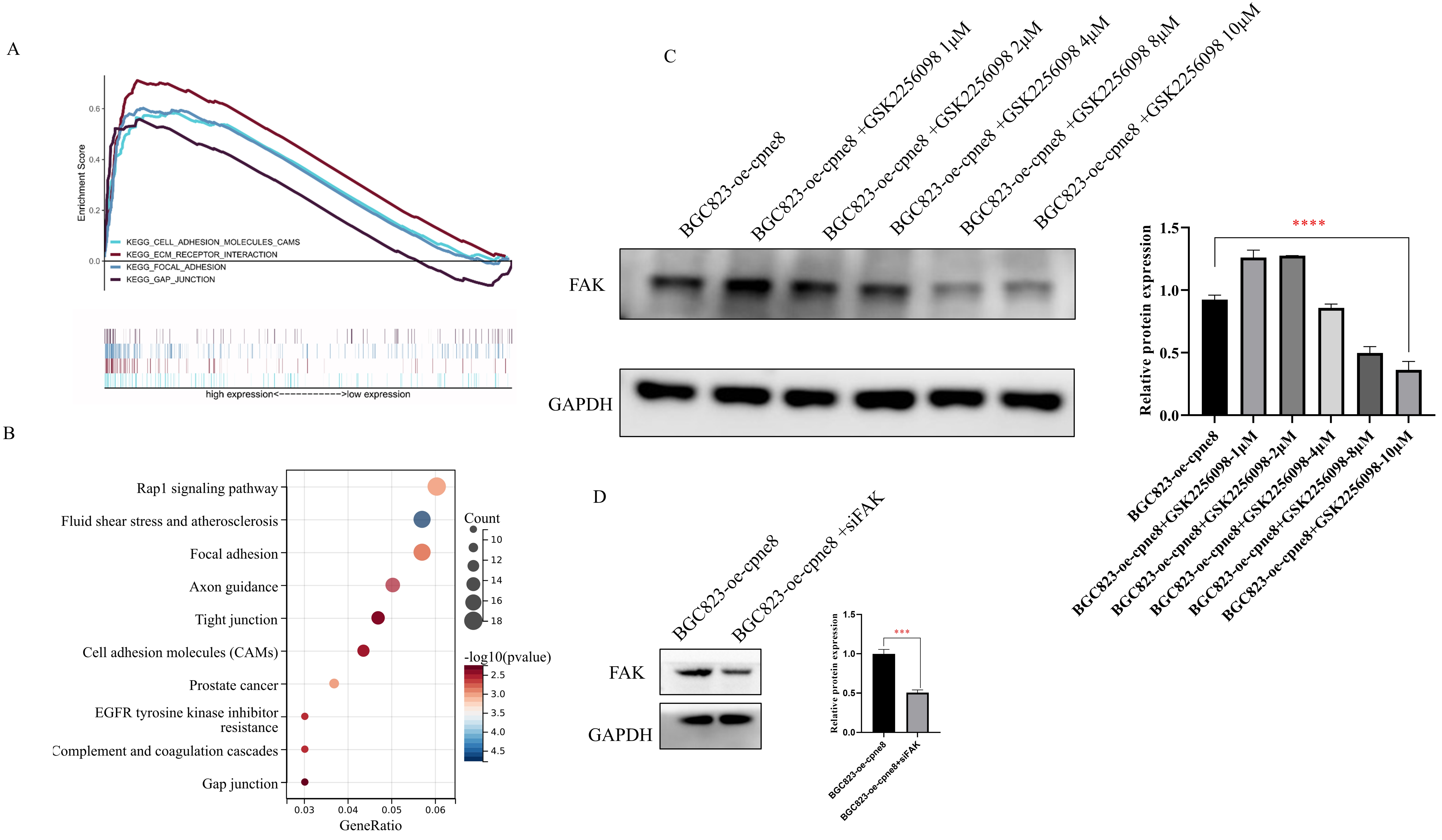
A
Enrichment Score
KEGG_CELL_ADHESION_MOLECULES_CAMS
KEGG_ECM_RECEPTOR_INTERACTION
KEGG_FOCAL_ADHESION
KEGG_GAP_JUNCTION
high expression<----------->low expression
B
Rap1 signaling pathway
Fluid shear stress and atherosclerosis
Focal adhesion
Axon guidance
Tight junction
Cell adhesion molecules (CAMs)
Prostate cancer
EGFR tyrosine kinase inhibitor resistance
Complement and coagulation cascades
Gap junction
Count
-log10(pvalue)
GeneRatio
C
BGC823-oe-cpne8
BGC823-oe-cpne8 +GSK2256098 1μM
BGC823-oe-cpne8 +GSK2256098 2μM
BGC823-oe-cpne8 +GSK2256098 4μM
BGC823-oe-cpne8 +GSK2256098 8μM
BGC823-oe-cpne8 +GSK2256098 10μM
FAK
GAPDH
Relative protein expression
****
BGC823-oe-cpne8+GSK2256098-1μM
BGC823-oe-cpne8+GSK2256098-2μM
BGC823-oe-cpne8+GSK2256098-4μM
BGC823-oe-cpne8+GSK2256098-8μM
BGC823-oe-cpne8+GSK2256098-10μM
D
BGC823-oe-cpne8 +siFAK
***
BGC823-oe-cpne8+siFAK

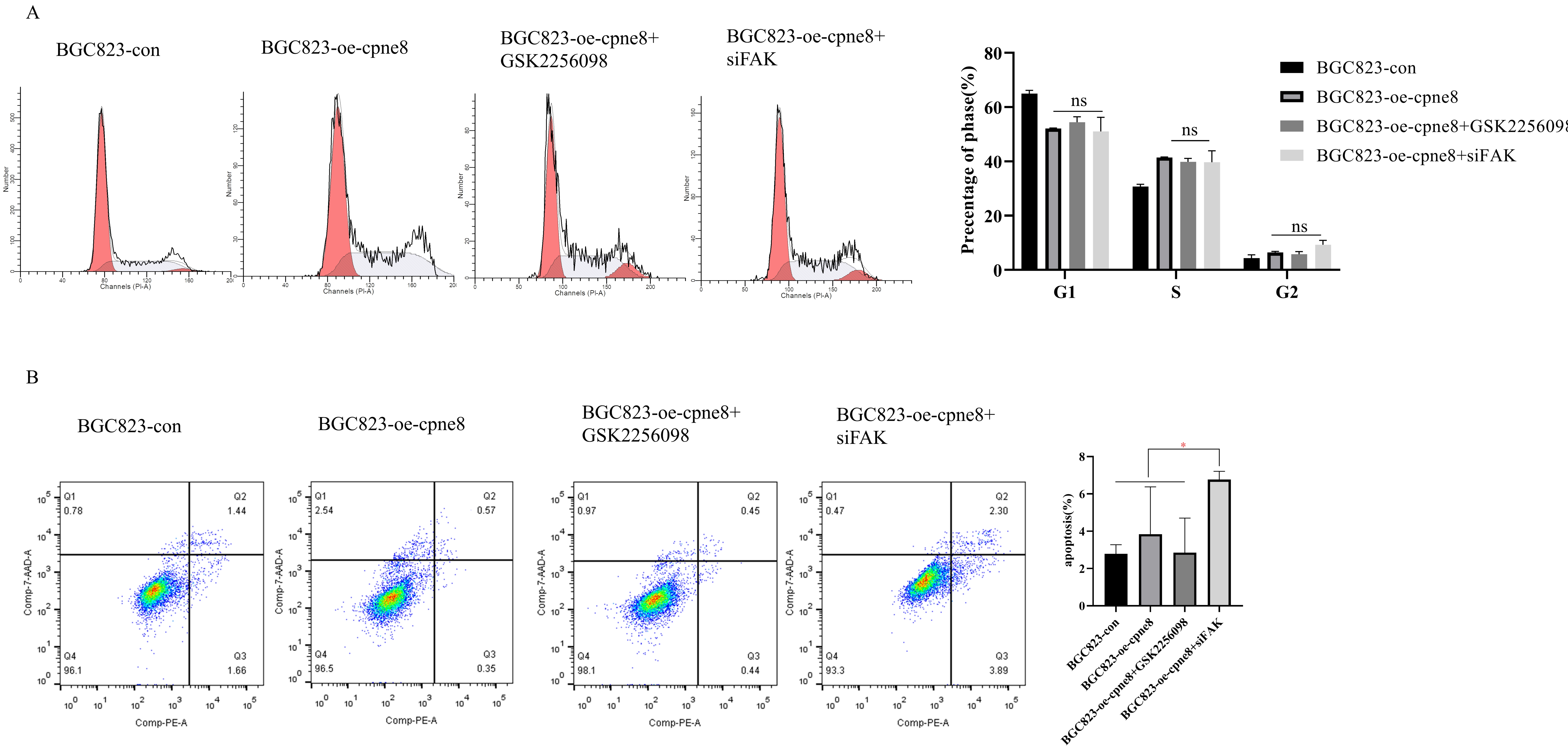
A
BGC823-con
BGC823-oe-cpne8
BGC823-oe-cpne8+
GSK2256098
BGC823-oe-cpne8+
siFAK
Number
Channels (PI-A)
Precentage of phase(%)
ns
G1
S
G2
BGC823-con
BGC823-oe-cpne8
BGC823-oe-cpne8+GSK225609
BGC823-oe-cpne8+siFAK
B
BGC823-con
BGC823-oe-cpne8
BGC823-oe-cpne8+
GSK2256098
BGC823-oe-cpne8+
siFAK
Q1 0.78
Q2 1.44
Q4 96.1
Q3 1.66
Q1 2.54
Q2 0.57
Q4 96.5
Q3 0.35
Q1 0.97
Q2 0.45
Q4 98.1
Q3 0.44
Q1 0.47
Q2 2.30
Q4 93.3
Q3 3.89
Comp-7-AAD-A
Comp-PE-A
apoptosis(%)
*
BGC823-con
BGC823-oe-cpne8
BGC823-oe-cpne8+GSK2256098
BGC823-oe-cpne8+siFAK

A

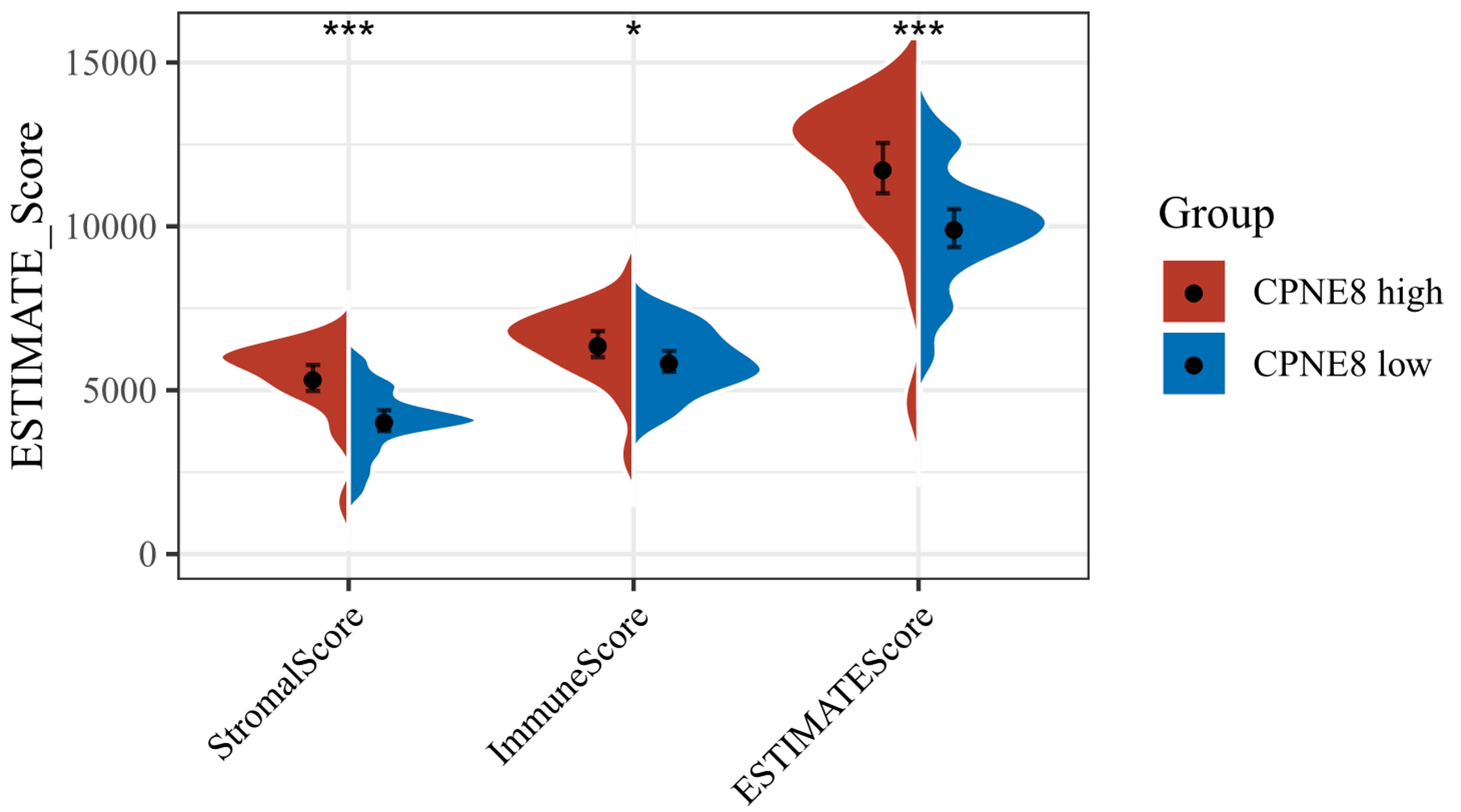

ESTIMATE_Score
15000
10000
5000
0
***
*
***
StromalScore
ImmuneScore
ESTIMATEScore
Group
CPNE8 high
CPNE8 low


B

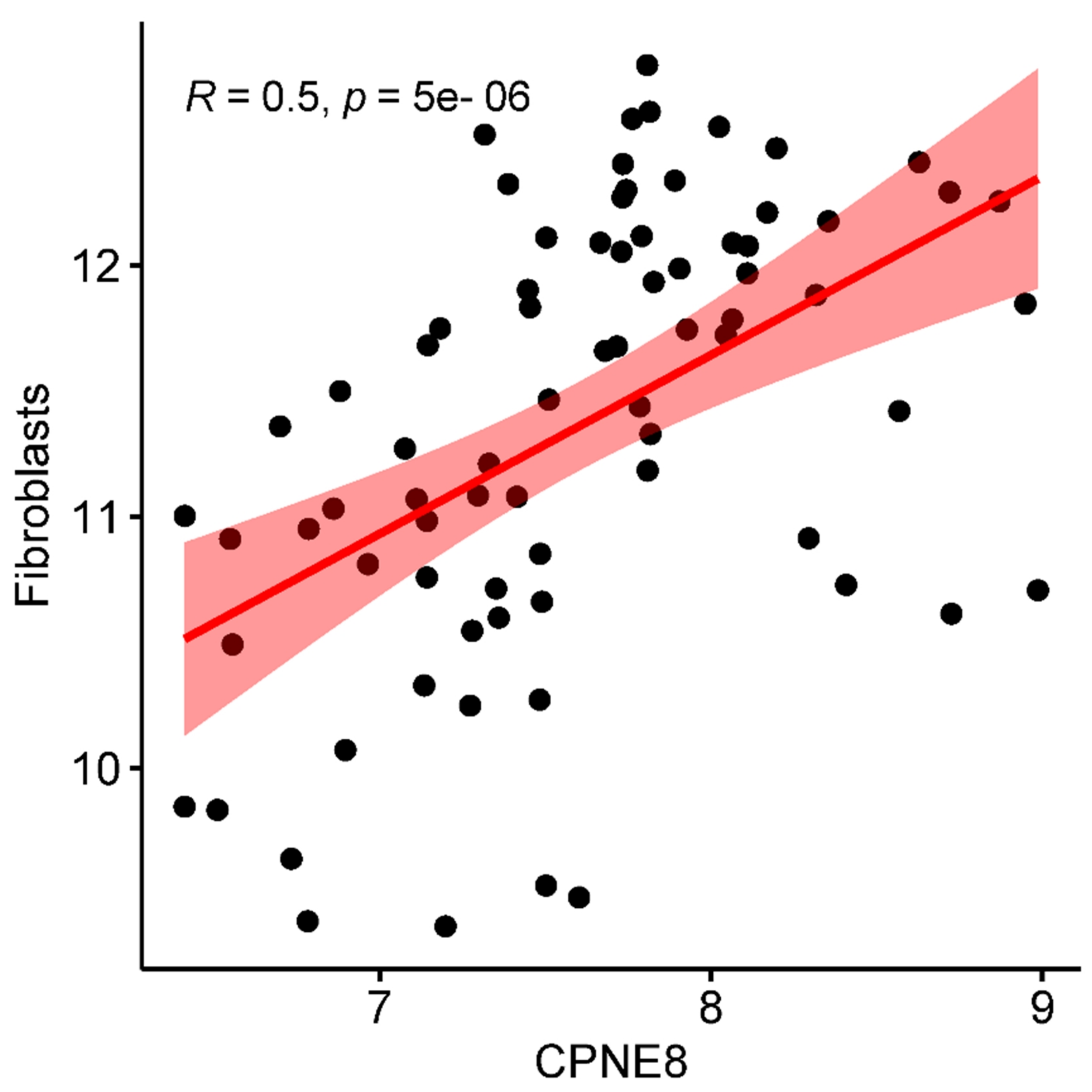

R = 0.5, p = 5e- 06
Fibroblasts
12
11
10
7
8
9
CPNE8

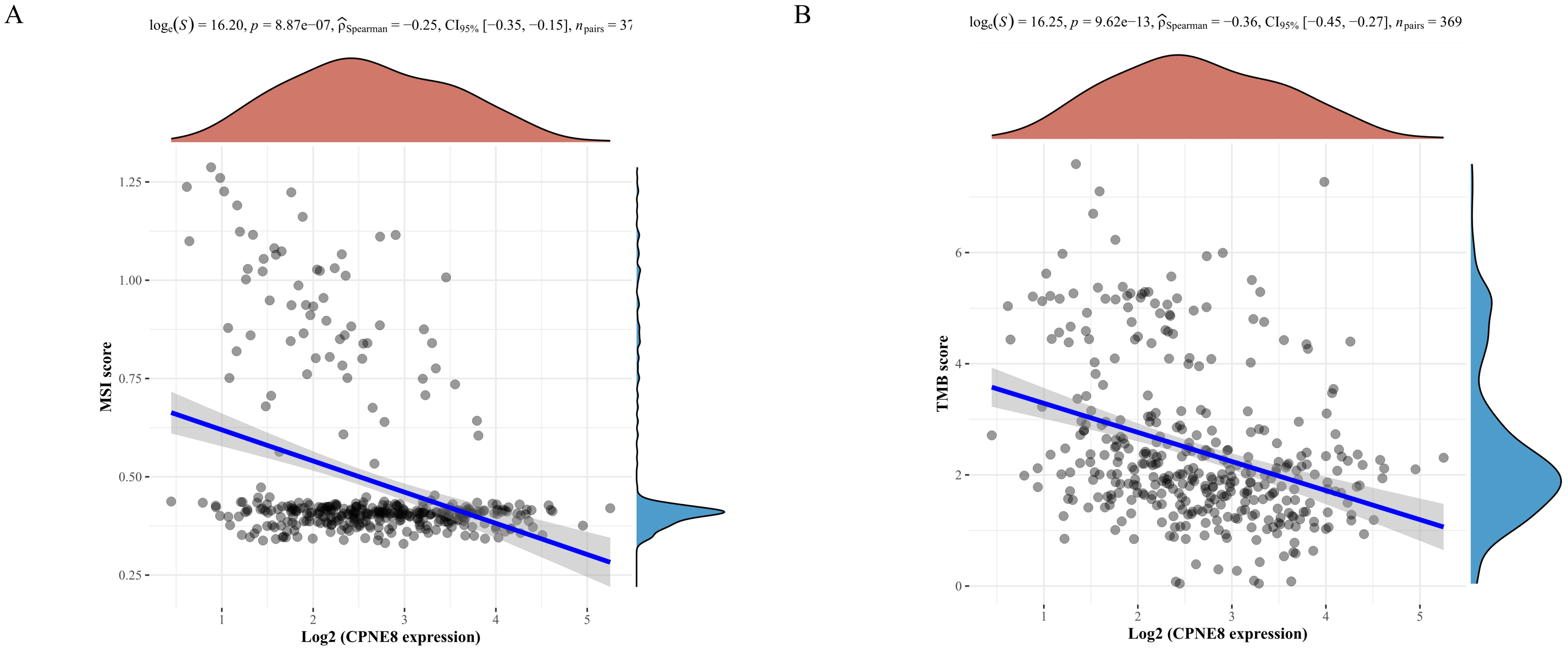
A
loge(S) = 16.20, p = 8.87e−07, ρ̂Spearman = −0.25, CI95% [−0.35, −0.15], npairs = 37
MSI score
Log2 (CPNE8 expression)
B
loge(S) = 16.25, p = 9.62e−13, ρ̂Spearman = −0.36, CI95% [−0.45, −0.27], npairs = 369
TMB score
Log2 (CPNE8 expression)

| ID | gender | grade | stage | T | M | N | Lymph node | AICC-edition |
|---|---|---|---|---|---|---|---|---|
| TCGA-B7-5818-01A-11R-1 | MALE | G3 | Stage I | T2 | M0 | N0 | 0 | 7th |
| TCGA-B7-A5TI-01A-11R-A | MALE | G3 | Stage III | T4 | M0 | N3 | 18 | 7th |
| TCGA-BR-4253-01A-01R-1 | FEMALE | G3 | Stage III | T3 | M0 | N1 | 3 | 6th |
| TCGA-BR-4267-01A-01R-1 | MALE | G2 | Stage I | T2 | M0 | N0 | 0 | 6th |
| TCGA-BR-4279-01A-01R-1 | MALE | G3 | Stage II | T2 | M0 | N1 | 3 | 6th |
| TCGA-BR-4280-01A-01R-1 | FEMALE | G2 | Stage III | T2 | M0 | N1 | 1 | 6th |
| TCGA-BR-4361-01A-01R-1 | FEMALE | G3 | Stage III | T4 | M0 | N0 | 0 | 6th |
| TCGA-BR-4368-01A-01R-1 | FEMALE | G3 | Stage IV | T4 | M0 | N2 | 8 | 6th |
| TCGA-BR-6452-01A-12R-1 | FEMALE | G3 | Stage II | T3 | M0 | N0 | 0 | 7th |
| TCGA-BR-6453-01A-11R-1 | MALE | G3 | Stage II | T2 | M0 | N1 | 1 | 7th |
| TCGA-BR-6454-01A-11R-1 | MALE | G3 | Stage II | T3 | M0 | N0 | 0 | 7th |
| TCGA-BR-6455-01A-11R-1 | MALE | G3 | Stage II | T3 | M0 | N1 | 2 | 7th |
| TCGA-BR-6456-01A-11R-1 | FEMALE | G2 | Stage II | T3 | M0 | N1 | 2 | 7th |
| TCGA-BR-6457-01A-21R-1 | MALE | G3 | Stage II | T3 | M0 | N0 | 0 | 7th |
| TCGA-BR-6458-01A-11R-1 | FEMALE | G3 | Stage II | T3 | M0 | N1 | 1 | 7th |
| TCGA-BR-6563-01A-13R-2 | MALE | G3 | Stage II | T3 | M0 | N1 | 1 | 7th |
| TCGA-BR-6564-01A-12R-1 | FEMALE | G3 | Stage III | T3 | M0 | N2 | 3 | 7th |
| TCGA-BR-6565-01A-11R-1 | MALE | G2 | Stage II | T4 | M0 | N0 | 0 | 7th |
| TCGA-BR-6566-01A-11R-1 | FEMALE | G3 | Stage II | T3 | M0 | N0 | 0 | 7th |
| TCGA-BR-6705-01A-12R-1 | FEMALE | G3 | Stage III | T3 | M0 | N3 | 9 | 7th |
| TCGA-BR-6707-01A-11R-1 | MALE | G3 | Stage II | T3 | M0 | N0 | 0 | 7th |
| TCGA-BR-6709-01A-11R-1 | FEMALE | G3 | Stage III | T3 | M0 | N3 | 15 | 7th |
| TCGA-BR-6710-01A-11R-1 | MALE | G2 | Stage I | T2 | M0 | N0 | 0 | 7th |
| TCGA-BR-6801-01A-11R-1 | MALE | G2 | Stage II | T3 | M0 | N0 | 0 | 7th |
| TCGA-BR-6802-01A-11R-1 | MALE | G3 | Stage III | T3 | M0 | N2 | 3 | 7th |
| TCGA-BR-6803-01A-11R-1 | FEMALE | G3 | Stage II | T3 | M0 | N0 | 0 | 7th |
| TCGA-BR-6852-01A-11R-1 | FEMALE | G3 | Stage II | T3 | M0 | N0 | 0 | 7th |
| TCGA-BR-7196-01A-11R-2 | MALE | G3 | Stage IV | T3 | M1 | N3 | 11 | 7th |
| TCGA-BR-7704-01A-11R-2 | FEMALE | G3 | Stage II | T3 | M0 | N0 | 0 | 7th |
| TCGA-BR-7707-01A-11R-2 | FEMALE | G3 | Stage I | T2 | M0 | N0 | 0 | 7th |
| TCGA-BR-7715-01A-11R-2 | MALE | G2 | Stage II | T3 | M0 | N0 | 0 | 7th |
| TCGA-BR-7716-01A-21R-2 | FEMALE | G2 | Stage II | T3 | M0 | N1 | 1 | 7th |
| TCGA-BR-7717-01A-11R-2 | MALE | G2 | Stage IV | T4 | M0 | N1 | 2 | 6th |
| TCGA-BR-7722-01A-31R-2 | MALE | G3 | Stage II | T3 | M0 | N1 | 5 | 7th |
| TCGA-BR-7723-01A-11R-2 | MALE | G3 | Stage III | T3 | M0 | N3 | 8 | 7th |
| TCGA-BR-7851-01A-11R-2 | MALE | G3 | Stage II | T4 | M0 | N0 | 0 | 7th |
| TCGA-BR-7901-01A-11R-2 | MALE | G2 | Stage II | T3 | M0 | N1 | 2 | 7th |
| TCGA-BR-7958-01A-21R-2 | MALE | G3 | Stage III | T4 | M0 | N0 | 0 | 7th |
| TCGA-BR-7959-01A-11R-2 | MALE | G3 | Stage III | T4 | M0 | N1 | 1 | 7th |
| TCGA-BR-8058-01A-31R-2 | FEMALE | G3 | Stage III | T4 | M0 | N2 | 3 | 7th |
| TCGA-BR-8059-01A-11R-2 | MALE | G3 | Stage III | T4 | M0 | N1 | 1 | 7th |
| TCGA-BR-8060-01A-11R-2 | FEMALE | G3 | Stage II | T2 | M0 | N2 | 4 | 7th |
| TCGA-BR-8077-01A-11R-2 | FEMALE | G2 | Stage III | T4 | M0 | N1 | 2 | 7th |
| TCGA-BR-8080-01A-11R-2 | FEMALE | G3 | Stage III | T4 | M0 | N3 | 10 | 7th |
| TCGA-BR-8081-01A-11R-2 | FEMALE | G2 | Stage II | T4 | M0 | N0 | 0 | 7th |
| TCGA-BR-8284-01A-11R-2 | FEMALE | G3 | Stage III | T4 | M0 | N3 | 9 | 7th |
| TCGA-BR-8286-01A-12R-2 | MALE | G3 | Stage II | T3 | M0 | N0 | 0 | 7th |
| TCGA-BR-8289-01A-11R-2 | MALE | G3 | Stage IV | T4 | M1 | N3 | 11 | 7th |
| TCGA-BR-8295-01A-11R-2 | FEMALE | G3 | Stage II | T4 | M0 | N0 | 0 | 7th |
| TCGA-BR-8296-01A-11R-2 | FEMALE | G3 | Stage III | T4 | M0 | N2 | 6 | 7th |
| TCGA-BR-8361-01A-11R-2 | FEMALE | G3 | Stage III | T4 | M0 | N2 | 6 | 7th |
| TCGA-BR-8364-01A-11R-2 | FEMALE | G3 | Stage III | T4 | M0 | N2 | 3 | 7th |
| TCGA-BR-8365-01A-21R-2 | FEMALE | G3 | Stage II | T3 | M0 | N0 | 0 | 7th |
| TCGA-BR-8366-01A-11R-2 | FEMALE | G3 | Stage II | T3 | M0 | N0 | 0 | 7th |
| TCGA-BR-8367-01A-11R-2 | MALE | G3 | Stage III | T3 | M0 | N3 | 9 | 7th |
| TCGA-BR-8368-01A-11R-2 | FEMALE | G3 | Stage I | T2 | M0 | N0 | 0 | 7th |
| TCGA-BR-8369-01A-11R-2 | FEMALE | G3 | Stage III | T3 | M0 | N3 | 7 | 7th |
| TCGA-BR-8371-01A-11R-2 | MALE | G3 | Stage III | T3 | M0 | N3 | 10 | 7th |
| TCGA-BR-8372-01A-11R-2 | MALE | G3 | Stage III | T4 | M0 | N3 | 8 | 7th |
| TCGA-BR-8373-01A-11R-2 | FEMALE | G2 | Stage III | T4 | M0 | N1 | 2 | 7th |
| TCGA-BR-8380-01A-11R-2 | MALE | G3 | Stage III | T4 | M0 | N3 | 11 | 7th |
| TCGA-BR-8381-01A-11R-2 | MALE | G3 | Stage II | T3 | M0 | N1 | 1 | 7th |
| TCGA-BR-8382-01A-11R-2 | FEMALE | G3 | Stage III | T4 | M0 | N3 | 9 | 7th |
| TCGA-BR-8384-01A-21R-2 | MALE | G3 | Stage III | T4 | M0 | N2 | 6 | 7th |
| TCGA-BR-8483-01A-31R-2 | MALE | G2 | Stage III | T3 | M0 | N2 | 4 | 7th |
| TCGA-BR-8484-01A-11R-2 | MALE | G2 | Stage III | T4 | M0 | N1 | 2 | 7th |
| TCGA-BR-8485-01A-11R-2 | FEMALE | G3 | Stage III | T4 | M0 | N3 | 7 | 7th |
| TCGA-BR-8486-01A-31R-2 | FEMALE | G2 | Stage I | T1 | M0 | N0 | 0 | 7th |
| TCGA-BR-8487-01A-11R-2 | FEMALE | G3 | Stage II | T3 | M0 | N0 | 0 | 7th |
| TCGA-BR-8588-01A-11R-2 | FEMALE | G3 | Stage II | T4 | M0 | N0 | 0 | 7th |
| TCGA-BR-8589-01A-11R-2 | MALE | G3 | Stage III | T4 | M0 | N1 | 1 | 7th |
| TCGA-BR-8590-01A-11R-2 | MALE | G3 | Stage III | T4 | M0 | N2 | 5 | 7th |
| TCGA-BR-8591-01A-11R-2 | MALE | G3 | Stage III | T4 | M0 | N3 | 14 | 7th |
| TCGA-BR-8592-01A-11R-2 | FEMALE | G3 | Stage III | T4 | M0 | N1 | 1 | 7th |
| TCGA-BR-8676-01A-11R-2 | MALE | G3 | Stage III | T3 | M0 | N3 | 10 | 7th |
| TCGA-BR-8677-01A-11R-2 | FEMALE | G3 | Stage III | T3 | M0 | N3 | 8 | 7th |
| TCGA-BR-8678-01A-11R-2 | MALE | G2 | Stage I | T2 | M0 | N0 | 0 | 7th |
| TCGA-BR-8679-01A-11R-2 | FEMALE | G2 | Stage I | T2 | M0 | N0 | 0 | 7th |
| TCGA-BR-8680-01A-11R-2 | MALE | G2 | Stage IV | T4 | M1 | N2 | 3 | 7th |
| TCGA-BR-8682-01A-11R-2 | MALE | G3 | Stage II | T4 | M0 | N0 | 0 | 7th |
| TCGA-BR-8683-01A-11R-2 | MALE | G3 | Stage III | T4 | M0 | N2 | 3 | 7th |
| TCGA-BR-8686-01A-11R-2 | MALE | G3 | Stage III | T4 | M0 | N1 | 2 | 7th |
| TCGA-BR-8687-01A-11R-2 | FEMALE | G3 | Stage III | T4 | M0 | N2 | 5 | 7th |
| TCGA-BR-8690-01A-11R-2 | FEMALE | G2 | Stage III | T3 | M0 | N3 | 10 | 7th |
| TCGA-BR-A44T-01A-32R- | FEMALE | G3 | Stage II | T3 | M0 | N0 | 0 | 7th |
| TCGA-BR-A44U-01A-11R- | MALE | G3 | Stage III | T3 | M0 | N3 | 10 | 7th |
| TCGA-BR-A4CR-01A-11R- | FEMALE | G3 | Stage III | T4 | M0 | N3 | 8 | 7th |
| TCGA-BR-A4CS-01A-11R- | MALE | G3 | Stage III | T4 | M0 | N3 | 7 | 7th |
| TCGA-BR-A4IV-01A-31R-A | MALE | G3 | Stage III | T4 | M0 | N2 | 3 | 7th |
| TCGA-BR-A4J4-01A-12R-A | MALE | G3 | Stage III | T4 | M0 | N2 | 3 | 7th |
| TCGA-BR-A4J5-01A-21R-A | MALE | G3 | Stage III | T4 | M0 | N1 | 1 | 7th |
| TCGA-BR-A4J6-01A-11R-A | FEMALE | G2 | Stage II | T3 | M0 | N0 | 0 | 7th |
| TCGA-BR-A4J8-01A-11R-A | FEMALE | G3 | Stage III | T3 | M0 | N3 | 12 | 7th |
| TCGA-BR-A4J9-01A-12R-A | MALE | G3 | Stage II | T3 | M0 | N0 | 0 | 7th |
| TCGA-BR-A4PF-01A-11R- | MALE | G3 | Stage III | T4 | M0 | N2 | 3 | 7th |
| TCGA-BR-A4QL-01A-31R- | FEMALE | G2 | Stage III | T3 | M0 | N3 | 9 | 7th |
| TCGA-CD-5798-01A-11R- | MALE | G2 | Stage II | T3 | M0 | N0 | 0 | 6th |
| TCGA-CD-5799-01A-11R- | MALE | G3 | Stage II | T2 | M0 | N1 | 0 | 6th |
| TCGA-CD-5800-01A-11R- | FEMALE | G1 | Stage II | T3 | M0 | N0 | 0 | 6th |
| TCGA-CD-5801-01A-11R- | MALE | G3 | Stage III | T3 | M0 | N1 | 0 | 6th |
| TCGA-CD-5803-01A-11R- | FEMALE | G3 | Stage II | T3 | M0 | N0 | 0 | 6th |
| TCGA-CD-5813-01A-11R- | MALE | G3 | Stage II | T3 | M0 | N0 | 0 | 6th |
| TCGA-CD-8524-01A-11R- | FEMALE | G3 | Stage II | T3 | M0 | N0 | 0 | 6th |
| TCGA-CD-8525-01A-11R- | FEMALE | G3 | Stage III | T3 | M0 | N1 | 2 | 6th |
| TCGA-CD-8526-01A-11R- | FEMALE | G3 | Stage III | T3 | M0 | N1 | 2 | 6th |
| TCGA-CD-8527-01A-11R- | FEMALE | G2 | Stage II | T2 | M0 | N1 | 0 | 6th |
| TCGA-CD-8528-01A-11R- | FEMALE | G3 | Stage III | T4 | M0 | N0 | 0 | 6th |
| TCGA-CD-8529-01A-11R- | MALE | G2 | Stage IV | T4 | M1 | N0 | 0 | 6th |
| TCGA-CD-8530-01A-11R- | MALE | G3 | Stage II | T3 | M0 | N0 | 0 | 6th |
| TCGA-CD-8531-01A-11R- | FEMALE | G3 | Stage III | T3 | M0 | N1 | 2 | 6th |
| TCGA-CD-8532-01A-11R- | MALE | G2 | Stage II | T3 | M0 | N0 | 0 | 6th |
| TCGA-CD-8533-01A-11R- | MALE | G3 | Stage II | T3 | M0 | N0 | 0 | 6th |
| TCGA-CD-8534-01A-11R- | MALE | G3 | Stage II | T3 | M0 | N0 | 0 | 6th |
| TCGA-CD-8535-01A-11R- | MALE | G3 | Stage III | T3 | M0 | N1 | 5 | 6th |
| TCGA-CD-A486-01A-11R- | MALE | G2 | Stage II | T3 | M0 | N0 | 0 | 7th |
| TCGA-CD-A487-01A-21R- | MALE | G3 | Stage II | T3 | M0 | N1 | 1 | 7th |
| TCGA-CD-A489-01A-11R- | MALE | G3 | Stage II | T3 | M0 | N0 | 0 | 7th |
| TCGA-CD-A48A-01A-12R- | MALE | G2 | Stage II | T3 | M0 | N0 | 0 | 7th |
| TCGA-CD-A48C-01A-11R- | FEMALE | G3 | Stage II | T3 | M0 | N1 | 0 | 7th |
| TCGA-CD-A4MG-01A-11R | MALE | G3 | Stage II | T3 | M0 | N0 | 0 | 7th |
| TCGA-CD-A4MH-01A-11R | FEMALE | G2 | Stage II | T3 | M0 | N0 | 0 | 7th |
| TCGA-CG-4301-01A-01R- | FEMALE | G3 | Stage IV | T4 | M1 | N1 | 6 | 5th |
| TCGA-CG-4304-01A-01R- | MALE | G2 | Stage I | T2 | M0 | N0 | 0 | 6th |
| TCGA-CG-4305-01A-01R- | MALE | G3 | Stage II | T2 | M0 | N1 | 2 | 6th |
| TCGA-CG-4306-01A-01R- | MALE | G3 | Stage IV | T3 | M1 | N2 | 0 | 5th |
| TCGA-CG-4436-01A-01R- | MALE | G2 | Stage I | T2 | M0 | N0 | 0 | 6th |
| TCGA-CG-4437-01A-01R- | MALE | G2 | Stage II | T2 | M0 | N1 | 3 | 5th |
| TCGA-CG-4438-01A-01R- | MALE | G3 | Stage IV | T4 | M0 | N2 | 14 | 5th |
| TCGA-CG-4440-01A-01R- | FEMALE | G3 | Stage IV | T3 | M1 | N3 | 18 | 5th |
| TCGA-CG-4441-01A-01R- | MALE | G2 | Stage III | T2 | M0 | N2 | 14 | 5th |
| TCGA-CG-4442-01A-01R- | MALE | G3 | Stage I | T2 | M0 | N0 | 0 | 5th |
| TCGA-CG-4443-01A-01R- | MALE | G2 | Stage I | T1 | M0 | N0 | 0 | 5th |
| TCGA-CG-4444-01A-01R- | MALE | G3 | Stage III | T2 | M0 | N2 | 15 | 6th |
| TCGA-CG-4460-01A-01R- | FEMALE | G2 | Stage IV | T4 | M1 | N1 | 2 | 6th |
| TCGA-CG-4462-01A-01R- | FEMALE | G3 | Stage IV | T3 | M1 | N3 | 25 | 6th |
| TCGA-CG-4465-01A-01R- | FEMALE | G3 | Stage IV | T4 | M1 | N3 | 21 | 6th |
| TCGA-CG-4466-01A-01R- | FEMALE | G2 | Stage I | T2 | M0 | N0 | 0 | 6th |
| TCGA-CG-4469-01A-01R- | MALE | G3 | Stage IV | T3 | M0 | N3 | 39 | 6th |
| TCGA-CG-4475-01A-01R- | MALE | G3 | Stage II | T3 | M0 | N1 | 2 | 7th |
| TCGA-CG-4476-01A-01R- | MALE | G3 | Stage III | T4 | M0 | N3 | 12 | 7th |
| TCGA-CG-4477-01A-01R- | FEMALE | G3 | Stage I | T2 | M0 | N0 | 0 | 6th |
| TCGA-CG-5716-01A-21R- | MALE | G2 | Stage IV | T4 | M1 | N2 | 0 | 7th |
| TCGA-CG-5717-01A-11R- | MALE | G3 | Stage II | T2 | M0 | N1 | 3 | 6th |
| TCGA-CG-5718-01A-11R- | FEMALE | G2 | Stage II | T2 | M0 | N1 | 3 | 6th |
| TCGA-CG-5719-01A-11R- | FEMALE | G1 | Stage IV | T4 | M1 | N0 | 0 | 7th |
| TCGA-CG-5720-01A-11R- | MALE | G3 | Stage I | T2 | M0 | N0 | 0 | 5th |
| TCGA-CG-5721-01A-11R- | MALE | G3 | Stage IV | T4 | M0 | N1 | 1 | 5th |
| TCGA-CG-5722-01A-21R- | FEMALE | G3 | Stage IV | T3 | M1 | N2 | 15 | 5th |
| TCGA-CG-5723-01A-11R- | MALE | G3 | Stage II | T2 | M0 | N1 | 1 | 5th |
| TCGA-CG-5724-01A-11R- | MALE | G3 | Stage IV | T3 | M1 | N3 | 34 | 5th |
| TCGA-CG-5725-01A-11R- | MALE | G3 | Stage I | T2 | M0 | N0 | 0 | 5th |
| TCGA-CG-5726-01A-11R- | MALE | G2 | Stage I | T1 | M0 | N0 | 0 | 5th |
| TCGA-CG-5732-01A-11R- | MALE | G2 | Stage IV | T2 | M0 | N3 | 17 | 5th |
| TCGA-CG-5734-01A-11R- | MALE | G3 | Stage III | T3 | M0 | N1 | 4 | 5th |
| TCGA-D7-5577-01A-01R- | FEMALE | G2 | Stage III | T2 | M0 | N3 | 19 | 7th |
| TCGA-D7-5578-01A-01R- | MALE | G3 | Stage III | T3 | M0 | N2 | 6 | 7th |
| TCGA-D7-6520-01A-11R- | MALE | G3 | Stage III | T2 | M0 | N2 | 14 | 6th |
| TCGA-D7-6522-01A-11R- | MALE | G3 | Stage I | T2 | M0 | N0 | 0 | 6th |
| TCGA-D7-6524-01A-11R- | MALE | G3 | Stage II | T2 | M0 | N1 | 6 | 6th |
| TCGA-D7-6525-01A-11R- | MALE | G3 | Stage III | T2 | M0 | N2 | 6 | 6th |
| TCGA-D7-6526-01A-11R- | FEMALE | G2 | Stage III | T3 | M0 | N2 | 3 | 7th |
| TCGA-D7-6527-01A-11R- | MALE | G2 | Stage II | T2 | M0 | N1 | 1 | 6th |
| TCGA-D7-6528-01A-11R- | FEMALE | G2 | Stage I | T2 | M0 | N0 | 0 | 6th |
| TCGA-D7-6815-01A-11R- | FEMALE | G2 | Stage II | T2 | M0 | N2 | 5 | 7th |
| TCGA-D7-6818-01A-11R- | MALE | G3 | Stage III | T2 | M0 | N3 | 11 | 7th |
| TCGA-D7-6822-01A-11R- | MALE | G2 | Stage I | T2 | M0 | N0 | 0 | 7th |
| TCGA-D7-8570-01A-11R-2 | MALE | G3 | Stage III | T3 | M0 | N3 | 13 | 7th |
| TCGA-D7-8572-01A-11R-2 | MALE | G2 | Stage II | T2 | M0 | N2 | 6 | 7th |
| TCGA-D7-8573-01A-11R-2 | MALE | G3 | Stage II | T3 | M0 | N0 | 0 | 7th |
| TCGA-D7-8574-01A-13R-2 | MALE | G3 | Stage III | T2 | M0 | N3 | 13 | 7th |
| TCGA-D7-8575-01A-11R-2 | MALE | G3 | Stage III | T3 | M0 | N2 | 6 | 7th |
| TCGA-D7-8576-01A-11R-2 | FEMALE | G3 | Stage III | T3 | M0 | N3 | 15 | 7th |
| TCGA-D7-8578-01A-21R-2 | MALE | G2 | Stage I | T2 | M0 | N0 | 0 | 7th |
| TCGA-D7-8579-01A-11R-2 | FEMALE | G2 | Stage II | T2 | M0 | N2 | 4 | 7th |
| TCGA-D7-A4YU-01A-21R- | MALE | G3 | Stage III | T3 | M0 | N3 | 25 | 7th |
| TCGA-D7-A4YX-01A-11R- | MALE | G3 | Stage II | T3 | M0 | N1 | 1 | 7th |
| TCGA-D7-A4Z0-01A-22R- | FEMALE | G3 | Stage II | T2 | M0 | N2 | 3 | 7th |
| TCGA-D7-A6EV-01A-11R- | FEMALE | G3 | Stage II | T2 | M0 | N2 | 3 | 7th |
| TCGA-D7-A6EX-01A-11R- | FEMALE | G3 | Stage III | T3 | M0 | N2 | 4 | 7th |
| TCGA-D7-A6EY-01A-21R- | FEMALE | G3 | Stage III | T3 | M0 | N3 | 13 | 7th |
| TCGA-D7-A6EZ-01A-11R- | MALE | G3 | Stage III | T3 | M0 | N2 | 3 | 7th |
| TCGA-D7-A6F0-01A-11R- | FEMALE | G3 | Stage I | T2 | M0 | N0 | 0 | 7th |
| TCGA-D7-A6F2-01A-12R- | MALE | G3 | Stage I | T2 | M0 | N0 | 0 | 7th |
| TCGA-D7-A747-01A-22R- | MALE | G3 | Stage II | T3 | M0 | N1 | 0 | 7th |
| TCGA-D7-A748-01A-12R- | FEMALE | G3 | Stage IV | T4 | M1 | N3 | 12 | 7th |
| TCGA-D7-A74A-01A-11R- | FEMALE | G2 | Stage III | T3 | M0 | N2 | 4 | 7th |
| TCGA-F1-6874-01A-11R-1 | MALE | G3 | Stage I | T2 | M0 | N0 | 0 | 7th |
| TCGA-F1-6875-01A-11R-2 | MALE | G2 | Stage I | T2 | M0 | N0 | 0 | 4th |
| TCGA-F1-A448-01A-11R-A | MALE | G3 | Stage III | T3 | M0 | N3 | 18 | 7th |
| TCGA-F1-A72C-01A-21R-A | MALE | G2 | Stage II | T3 | M0 | N0 | 0 | 7th |
| TCGA-FP-7829-01A-11R-2 | MALE | G2 | Stage II | T3 | M0 | N1 | 2 | 7th |
| TCGA-FP-8209-01A-11R-2 | MALE | G3 | Stage I | T2 | M0 | N0 | 0 | 6th |
| TCGA-FP-8210-01A-11R-2 | MALE | G3 | Stage III | T3 | M0 | N1 | 3 | 6th |
| TCGA-FP-A4BF-01A-12R-A | MALE | G3 | Stage III | T3 | M0 | N2 | 5 | 7th |
| TCGA-HF-7132-01A-11R-2 | MALE | G2 | Stage II | T2 | M0 | N1 | 2 | 6th |
| TCGA-HF-7133-01A-11R-2 | FEMALE | G2 | Stage IV | T2 | M0 | N3 | 16 | 6th |
| TCGA-HF-7134-01A-11R-2 | MALE | G2 | Stage I | T1 | M0 | N0 | 0 | 6th |
| TCGA-HF-A5NB-01A-11R- | FEMALE | G3 | Stage III | T4 | M0 | N3 | 11 | 7th |
| TCGA-HU-8244-01A-11R- | FEMALE | G1 | Stage I | T1 | M0 | N0 | 0 | 7th |
| TCGA-HU-8249-01A-11R- | MALE | G2 | Stage III | T3 | M0 | N2 | 5 | 7th |
| TCGA-HU-8602-01A-11R- | FEMALE | G3 | Stage II | T4 | M0 | N0 | 0 | 7th |
| TCGA-HU-8604-01A-11R- | FEMALE | G2 | Stage II | T3 | M0 | N0 | 0 | 7th |
| TCGA-HU-8608-01A-11R- | MALE | G3 | Stage III | T4 | M0 | N2 | 4 | 7th |
| TCGA-HU-8610-01A-22R- | MALE | G2 | Stage I | T1 | M0 | N0 | 0 | 7th |
| TCGA-HU-A4G2-01A-11R- | MALE | G3 | Stage II | T3 | M0 | N1 | 2 | 7th |
| TCGA-HU-A4G3-01A-11R- | MALE | G2 | Stage II | T2 | M0 | N2 | 5 | 7th |
| TCGA-HU-A4G8-01A-11R- | FEMALE | G3 | Stage II | T3 | M0 | N1 | 1 | 7th |
| TCGA-HU-A4G9-01A-11R- | FEMALE | G2 | Stage I | T1 | M0 | N0 | 0 | 7th |
| TCGA-HU-A4GC-01A-12R- | MALE | G3 | Stage III | T4 | M0 | N2 | 3 | 7th |
| TCGA-HU-A4GD-01A-11R | MALE | G3 | Stage II | T3 | M0 | N1 | 1 | 7th |
| TCGA-HU-A4GF-01A-11R- | MALE | G2 | Stage II | T3 | M0 | N0 | 0 | 7th |
| TCGA-HU-A4GH-01A-11R | MALE | G2 | Stage I | T1 | M0 | N0 | 0 | 7th |
| TCGA-HU-A4GJ-01A-11R- | FEMALE | G3 | Stage III | T4 | M0 | N3 | 14 | 7th |
| TCGA-HU-A4GP-01A-11R- | FEMALE | G2 | Stage II | T2 | M0 | N1 | 2 | 7th |
| TCGA-HU-A4GQ-01A-11R | MALE | G2 | Stage III | T4 | M0 | N2 | 4 | 7th |
| TCGA-HU-A4GT-01A-21R- | FEMALE | G3 | Stage II | T3 | M0 | N0 | 0 | 7th |
| TCGA-HU-A4GU-01A-11R | MALE | G3 | Stage II | T3 | M0 | N1 | 2 | 7th |
| TCGA-HU-A4GX-01A-12R- | FEMALE | G3 | Stage III | T4 | M0 | N2 | 6 | 7th |
| TCGA-HU-A4GY-01A-21R- | FEMALE | G3 | Stage III | T4 | M0 | N1 | 1 | 7th |
| TCGA-HU-A4H0-01A-11R- | MALE | G3 | Stage III | T4 | M0 | N3 | 49 | 7th |
| TCGA-HU-A4H2-01A-11R- | FEMALE | G2 | Stage III | T3 | M0 | N3 | 34 | 7th |
| TCGA-HU-A4H3-01A-21R- | FEMALE | G3 | Stage III | T4 | M0 | N3 | 16 | 7th |
| TCGA-HU-A4H4-01A-21R- | FEMALE | G3 | Stage II | T2 | M0 | N2 | 4 | 7th |
| TCGA-HU-A4H6-01A-11R- | FEMALE | G2 | Stage III | T3 | M0 | N2 | 3 | 7th |
| TCGA-HU-A4H8-01A-11R- | MALE | G2 | Stage I | T1 | M0 | N1 | 1 | 7th |
| TCGA-HU-A4HB-01A-12R- | MALE | G3 | Stage II | T2 | M0 | N2 | 5 | 7th |
| TCGA-HU-A4HD-01A-11R | MALE | G2 | Stage III | T3 | M0 | N2 | 3 | 7th |
| TCGA-IN-7806-01A-11R-2 | MALE | G3 | Stage II | T3 | M0 | N1 | 2 | 7th |
| TCGA-IN-8462-01A-11R-2 | MALE | G2 | Stage II | T2 | M0 | N1 | 2 | 7th |
| TCGA-IN-8663-01A-11R-2 | MALE | G3 | Stage II | T2 | M0 | N2 | 9 | 7th |
| TCGA-IN-A6RI-01A-11R-A | MALE | G2 | Stage I | T1 | M0 | N0 | 0 | 7th |
| TCGA-IN-A6RJ-01A-21R-A | MALE | G3 | Stage I | T1 | M0 | N0 | 0 | 7th |
| TCGA-IN-A6RN-01A-12R- | FEMALE | G2 | Stage II | T1 | M0 | N2 | 9 | 7th |
| TCGA-IN-A6RO-01A-12R- | MALE | G2 | Stage I | T1 | M0 | N0 | 0 | 7th |
| TCGA-IN-A6RS-01A-12R-A | MALE | G2 | Stage I | T1 | M0 | N0 | 0 | 7th |
| TCGA-IN-A7NR-01A-11R- | FEMALE | G3 | Stage IV | T3 | M1 | N3 | 9 | 7th |
| TCGA-IN-A7NT-01A-21R- | FEMALE | G3 | Stage II | T3 | M0 | N1 | 1 | 7th |
| TCGA-IN-A7NU-01A-22R- | MALE | G3 | Stage III | T3 | M0 | N3 | 12 | 7th |
| TCGA-IN-AB1V-01A-21R-A | MALE | G2 | Stage I | T1 | M0 | N0 | 0 | 7th |
| TCGA-IN-AB1X-01A-11R-A | FEMALE | G3 | Stage II | T3 | M0 | N0 | 0 | 7th |
| TCGA-KB-A6F7-01A-12R-A | FEMALE | G3 | Stage I | T1 | M0 | N1 | 1 | 6th |
| TCGA-KB-A93G-01A-11R- | MALE | G1 | Stage I | T2 | M0 | N0 | 0 | 7th |
| TCGA-KB-A93H-01A-11R- | FEMALE | G1 | Stage II | T3 | M0 | N1 | 1 | 7th |
| TCGA-KB-A93J-01A-11R-A | MALE | G3 | Stage II | T2 | M0 | N1 | 1 | 6th |
| TCGA-MX-A5UG-01A-21R | MALE | G3 | Stage III | T3 | M0 | N1 | 2 | 6th |
| TCGA-MX-A5UJ-01A-11R- | FEMALE | G3 | Stage III | T3 | M0 | N2 | 6 | 7th |
| TCGA-MX-A663-01A-11R- | MALE | G3 | Stage II | T3 | M0 | N0 | 0 | 7th |
| TCGA-R5-A7O7-01A-11R- | MALE | G2 | Stage IV | T3 | M1 | N1 | 0 | 7th |
| TCGA-R5-A7ZE-01B-11R-A | FEMALE | G2 | Stage III | T3 | M0 | N2 | 0 | 7th |
| TCGA-R5-A7ZF-01A-11R-A | FEMALE | G2 | Stage IV | T4 | M1 | N1 | 0 | 7th |
| TCGA-R5-A7ZI-01A-11R-A | FEMALE | G3 | Stage IV | T4 | M1 | N1 | 0 | 6th |
| TCGA-R5-A805-01A-11R-A | MALE | G3 | Stage III | T3 | M0 | N2 | 0 | 6th |
| TCGA-RD-A7BS-01A-11R- | MALE | G3 | Stage III | T3 | M0 | N1 | 5 | 6th |
| TCGA-RD-A7BT-01A-11R- | MALE | G3 | Stage IV | T3 | M0 | N3 | 55 | 6th |
| TCGA-RD-A7BW-01A-11R | FEMALE | G3 | Stage I | T2 | M0 | N0 | 0 | 6th |
| TCGA-RD-A7C1-01A-11R- | MALE | G3 | Stage I | T2 | M0 | N0 | 0 | 6th |
| TCGA-RD-A8MV-01A-11R | MALE | G3 | Stage III | T3 | M0 | N2 | 9 | 6th |
| TCGA-RD-A8MW-01A-11R | MALE | G3 | Stage III | T3 | M0 | N2 | 11 | 6th |
| TCGA-RD-A8N0-01A-12R- | FEMALE | G3 | Stage III | T3 | M0 | N2 | 10 | 6th |
| TCGA-RD-A8N1-01A-12R- | MALE | G3 | Stage III | T3 | M0 | N2 | 10 | 6th |
| TCGA-RD-A8N2-01A-12R- | FEMALE | G3 | Stage I | T2 | M0 | N0 | 0 | 6th |
| TCGA-RD-A8N4-01A-21R- | FEMALE | G3 | Stage III | T3 | M0 | N1 | 3 | 6th |
| TCGA-RD-A8N5-01A-12R- | MALE | G2 | Stage III | T3 | M0 | N1 | 4 | 6th |
| TCGA-RD-A8N6-01A-11R- | FEMALE | G3 | Stage III | T2 | M0 | N2 | 10 | 6th |
| TCGA-RD-A8N9-01A-12R- | FEMALE | G3 | Stage II | T2 | M0 | N1 | 2 | 6th |
| TCGA-RD-A8NB-01A-12R- | FEMALE | G3 | Stage III | T3 | M0 | N1 | 3 | 6th |
| TCGA-SW-A7EB-01A-11R- | MALE | G2 | Stage III | T3 | M0 | N2 | 3 | 7th |
| TCGA-VQ-A8DT-01A-11R- | MALE | G3 | Stage III | T3 | M0 | N3 | 9 | 7th |
| TCGA-VQ-A8DU-01A-11R | MALE | G2 | Stage III | T3 | M0 | N2 | 8 | 7th |
| TCGA-VQ-A8DV-01A-12R | MALE | G2 | Stage I | T2 | M0 | N0 | 0 | 6th |
| TCGA-VQ-A8DZ-01A-11R | MALE | G3 | Stage IV | T4 | M1 | N3 | 18 | 7th |
| TCGA-VQ-A8E0-01A-11R- | MALE | G3 | Stage III | T3 | M0 | N2 | 6 | 7th |
| TCGA-VQ-A8E2-01A-11R- | MALE | G2 | Stage III | T3 | M0 | N2 | 8 | 6th |
| TCGA-VQ-A8E3-01A-11R- | MALE | G3 | Stage II | T3 | M0 | N0 | 0 | 7th |
| TCGA-VQ-A8E7-01B-11R- | MALE | G2 | Stage IV | T3 | M1 | N1 | 1 | 7th |
| TCGA-VQ-A8P2-01A-11R- | MALE | G2 | Stage III | T4 | M0 | N1 | 1 | 7th |
| TCGA-VQ-A8P3-01A-11R- | MALE | G2 | Stage III | T4 | M0 | N1 | 1 | 7th |
| TCGA-VQ-A8P5-01A-11R- | MALE | G3 | Stage II | T3 | M0 | N0 | 0 | 7th |
| TCGA-VQ-A8P8-01A-11R- | FEMALE | G2 | Stage II | T4 | M0 | N0 | 0 | 7th |
| TCGA-VQ-A8PB-01A-11R- | FEMALE | G2 | Stage II | T3 | M0 | N0 | 0 | 6th |
| TCGA-VQ-A8PC-01A-11R- | MALE | G3 | Stage III | T3 | M0 | N1 | 2 | 6th |
| TCGA-VQ-A8PD-01A-11R | MALE | G2 | Stage III | T4 | M0 | N3 | 26 | 7th |
| TCGA-VQ-A8PE-01A-11R- | MALE | G3 | Stage III | T3 | M0 | N3 | 12 | 7th |
| TCGA-VQ-A8PF-01A-11R- | MALE | G3 | Stage III | T3 | M0 | N3 | 24 | 7th |
| TCGA-VQ-A8PH-01A-12R | MALE | G2 | Stage III | T3 | M0 | N3 | 24 | 7th |
| TCGA-VQ-A8PK-01A-12R- | MALE | G2 | Stage III | T3 | M0 | N3 | 11 | 7th |
| TCGA-VQ-A8PM-01A-21R | MALE | G3 | Stage IV | T4 | M0 | N3 | 55 | 6th |
| TCGA-VQ-A8PO-01A-11R | MALE | G3 | Stage II | T4 | M0 | N0 | 0 | 7th |
| TCGA-VQ-A8PP-01A-21R- | MALE | G2 | Stage IV | T4 | M0 | N1 | 3 | 6th |
| TCGA-VQ-A8PQ-01A-11R | FEMALE | G2 | Stage IV | T4 | M1 | N1 | 6 | 6th |
| TCGA-VQ-A8PU-01A-12R | FEMALE | G3 | Stage III | T4 | M0 | N1 | 1 | 7th |
| TCGA-VQ-A8PX-01A-12R- | MALE | G2 | Stage I | T1 | M0 | N0 | 0 | 6th |
| TCGA-VQ-A91A-01A-11R- | MALE | G1 | Stage III | T3 | M0 | N3 | 11 | 7th |
| TCGA-VQ-A91D-01A-11R | MALE | G2 | Stage III | T4 | M0 | N2 | 5 | 7th |
| TCGA-VQ-A91E-01A-31R- | FEMALE | G3 | Stage III | T4 | M0 | N0 | 0 | 7th |
| TCGA-VQ-A91K-01A-11R- | MALE | G2 | Stage III | T3 | M0 | N1 | 4 | 6th |
| TCGA-VQ-A91N-01A-11R | FEMALE | G2 | Stage IV | T4 | M0 | N3 | 18 | 6th |
| TCGA-VQ-A91Q-01A-12R | MALE | G3 | Stage IV | T3 | M0 | N3 | 25 | 6th |
| TCGA-VQ-A91U-01A-11R- | MALE | G2 | Stage III | T3 | M0 | N1 | 2 | 6th |
| TCGA-VQ-A91V-01A-11R- | MALE | G2 | Stage III | T3 | M0 | N2 | 4 | 7th |
| TCGA-VQ-A91X-01A-12R- | MALE | G3 | Stage III | T3 | M0 | N2 | 14 | 6th |
| TCGA-VQ-A91Y-01A-11R- | MALE | G3 | Stage III | T4 | M0 | N3 | 50 | 6th |
| TCGA-VQ-A91Z-01A-11R- | FEMALE | G2 | Stage III | T3 | M0 | N1 | 2 | 6th |
| TCGA-VQ-A922-01A-11R- | MALE | G2 | Stage IV | T4 | M0 | N1 | 1 | 6th |
| TCGA-VQ-A923-01A-11R- | MALE | G3 | Stage III | T3 | M0 | N1 | 4 | 6th |
| TCGA-VQ-A924-01A-11R- | MALE | G2 | Stage II | T3 | M0 | N0 | 0 | 6th |
| TCGA-VQ-A925-01A-11R- | MALE | G2 | Stage III | T3 | M0 | N2 | 6 | 7th |
| TCGA-VQ-A927-01A-12R- | MALE | G1 | Stage III | T3 | M0 | N3 | 27 | 7th |
| TCGA-VQ-A928-01A-11R- | MALE | G2 | Stage IV | T3 | M0 | N3 | 32 | 6th |
| TCGA-VQ-A92D-01A-11R | MALE | G2 | Stage I | T2 | M0 | N0 | 0 | 6th |
| TCGA-VQ-A94O-01A-11R | MALE | G2 | Stage III | T4 | M0 | N3 | 18 | 7th |
| TCGA-VQ-A94T-01A-11R- | MALE | G3 | Stage III | T3 | M0 | N2 | 9 | 6th |
| TCGA-VQ-A94U-01A-12R- | MALE | G3 | Stage II | T4 | M0 | N0 | 0 | 7th |
| TCGA-VQ-AA64-01A-11R- | MALE | G2 | Stage III | T3 | M0 | N2 | 6 | 7th |
| TCGA-VQ-AA68-01A-11R- | FEMALE | G2 | Stage III | T4 | M0 | N3 | 21 | 7th |
| TCGA-VQ-AA69-01A-11R- | MALE | G2 | Stage III | T3 | M0 | N2 | 6 | 7th |
| TCGA-VQ-AA6A-01A-11R | MALE | G2 | Stage III | T4 | M0 | N3 | 8 | 7th |
| TCGA-VQ-AA6D-01A-11R | FEMALE | G2 | Stage III | T4 | M0 | N1 | 2 | 7th |
| TCGA-VQ-AA6F-01A-31R- | MALE | G2 | Stage II | T3 | M0 | N1 | 1 | 7th |
| TCGA-VQ-AA6G-01A-11R | MALE | G2 | Stage II | T3 | M0 | N0 | 0 | 7th |
| TCGA-VQ-AA6J-01A-11R- | MALE | G3 | Stage III | T4 | M0 | N2 | 3 | 7th |
| TCGA-VQ-AA6K-01A-11R- | MALE | G3 | Stage III | T4 | M0 | N3 | 10 | 6th |

| ID | gender | grade | Stage | T | M | N | corrected |
|---|---|---|---|---|---|---|---|
| TCGA-B7-5818-01A-11R-16 | MALE | G3 | Stage I | T2 | M0 | N0 | 7th |
| TCGA-B7-A5TI-01A-11R-A3 | MALE | G3 | Stage III | T4 | M0 | N3 | 7th |
| TCGA-BR-4253-01A-01R-11 | FEMALE | G3 | Stage III | T3 | M0 | N2 | 7th |
| TCGA-BR-4267-01A-01R-11 | MALE | G2 | Stage I | T2 | M0 | N0 | 7th |
| TCGA-BR-4279-01A-01R-11 | MALE | G3 | Stage II | T2 | M0 | N2 | 7th |
| TCGA-BR-4280-01A-01R-11 | FEMALE | G2 | Stage II | T3 | M0 | N1 | 7th |
| TCGA-BR-4361-01A-01R-11 | FEMALE | G3 | Stage III | T4 | M0 | N0 | 7th |
| TCGA-BR-4368-01A-01R-11 | FEMALE | G3 | Stage III | T4 | M0 | N3 | 7th |
| TCGA-BR-6452-01A-12R-18 | FEMALE | G3 | Stage II | T3 | M0 | N0 | 7th |
| TCGA-BR-6453-01A-11R-18 | MALE | G3 | Stage II | T2 | M0 | N1 | 7th |
| TCGA-BR-6454-01A-11R-18 | MALE | G3 | Stage II | T3 | M0 | N0 | 7th |
| TCGA-BR-6455-01A-11R-18 | MALE | G3 | Stage II | T3 | M0 | N1 | 7th |
| TCGA-BR-6456-01A-11R-18 | FEMALE | G2 | Stage II | T3 | M0 | N1 | 7th |
| TCGA-BR-6457-01A-21R-18 | MALE | G3 | Stage II | T3 | M0 | N0 | 7th |
| TCGA-BR-6458-01A-11R-18 | FEMALE | G3 | Stage II | T3 | M0 | N1 | 7th |
| TCGA-BR-6563-01A-13R-20 | MALE | G3 | Stage II | T3 | M0 | N1 | 7th |
| TCGA-BR-6564-01A-12R-18 | FEMALE | G3 | Stage III | T3 | M0 | N2 | 7th |
| TCGA-BR-6565-01A-11R-18 | MALE | G2 | Stage II | T4 | M0 | N0 | 7th |
| TCGA-BR-6566-01A-11R-18 | FEMALE | G3 | Stage II | T3 | M0 | N0 | 7th |
| TCGA-BR-6705-01A-12R-18 | FEMALE | G3 | Stage III | T3 | M0 | N3 | 7th |
| TCGA-BR-6707-01A-11R-18 | MALE | G3 | Stage II | T3 | M0 | N0 | 7th |
| TCGA-BR-6709-01A-11R-18 | FEMALE | G3 | Stage III | T3 | M0 | N3 | 7th |
| TCGA-BR-6710-01A-11R-18 | MALE | G2 | Stage I | T2 | M0 | N0 | 7th |
| TCGA-BR-6801-01A-11R-18 | MALE | G2 | Stage II | T3 | M0 | N0 | 7th |
| TCGA-BR-6802-01A-11R-18 | MALE | G3 | Stage III | T3 | M0 | N2 | 7th |
| TCGA-BR-6803-01A-11R-18 | FEMALE | G3 | Stage II | T3 | M0 | N0 | 7th |
| TCGA-BR-6852-01A-11R-18 | FEMALE | G3 | Stage II | T3 | M0 | N0 | 7th |
| TCGA-BR-7196-01A-11R-20 | MALE | G3 | Stage IV | T3 | M1 | N3 | 7th |
| TCGA-BR-7704-01A-11R-20 | FEMALE | G3 | Stage II | T3 | M0 | N0 | 7th |
| TCGA-BR-7707-01A-11R-20 | FEMALE | G3 | Stage I | T2 | M0 | N0 | 7th |
| TCGA-BR-7715-01A-11R-20 | MALE | G2 | Stage II | T3 | M0 | N0 | 7th |
| TCGA-BR-7716-01A-21R-20 | FEMALE | G2 | Stage II | T3 | M0 | N1 | 7th |
| TCGA-BR-7717-01A-11R-20 | MALE | G2 | Stage III | T4 | M0 | N1 | 7th |
| TCGA-BR-7722-01A-31R-22 | MALE | G3 | Stage II | T3 | M0 | N1 | 7th |
| TCGA-BR-7723-01A-11R-20 | MALE | G3 | Stage III | T3 | M0 | N3 | 7th |
| TCGA-BR-7851-01A-11R-22 | MALE | G3 | Stage II | T4 | M0 | N0 | 7th |
| TCGA-BR-7901-01A-11R-22 | MALE | G2 | Stage II | T3 | M0 | N1 | 7th |
| TCGA-BR-7958-01A-21R-23 | MALE | G3 | Stage III | T4 | M0 | N0 | 7th |
| TCGA-BR-7959-01A-11R-23 | MALE | G3 | Stage III | T4 | M0 | N1 | 7th |
| TCGA-BR-8058-01A-31R-23 | FEMALE | G3 | Stage III | T4 | M0 | N2 | 7th |
| TCGA-BR-8059-01A-11R-23 | MALE | G3 | Stage III | T4 | M0 | N1 | 7th |
| TCGA-BR-8060-01A-11R-23 | FEMALE | G3 | Stage II | T2 | M0 | N2 | 7th |
| TCGA-BR-8077-01A-11R-23 | FEMALE | G2 | Stage III | T4 | M0 | N1 | 7th |
| TCGA-BR-8080-01A-11R-23 | FEMALE | G3 | Stage III | T4 | M0 | N3 | 7th |
| TCGA-BR-8081-01A-11R-23 | FEMALE | G2 | Stage II | T4 | M0 | N0 | 7th |
| TCGA-BR-8284-01A-11R-23 | FEMALE | G3 | Stage III | T4 | M0 | N3 | 7th |
| TCGA-BR-8286-01A-12R-23 | MALE | G3 | Stage II | T3 | M0 | N0 | 7th |
| TCGA-BR-8289-01A-11R-23 | MALE | G3 | Stage IV | T4 | M1 | N3 | 7th |
| TCGA-BR-8295-01A-11R-23 | FEMALE | G3 | Stage II | T4 | M0 | N0 | 7th |
| TCGA-BR-8296-01A-11R-23 | FEMALE | G3 | Stage III | T4 | M0 | N2 | 7th |
| TCGA-BR-8361-01A-11R-23 | FEMALE | G3 | Stage III | T4 | M0 | N2 | 7th |
| TCGA-BR-8364-01A-11R-23 | FEMALE | G3 | Stage III | T4 | M0 | N2 | 7th |
| TCGA-BR-8365-01A-21R-23 | FEMALE | G3 | Stage II | T3 | M0 | N0 | 7th |
| TCGA-BR-8366-01A-11R-23 | FEMALE | G3 | Stage II | T3 | M0 | N0 | 7th |
| TCGA-BR-8367-01A-11R-23 | MALE | G3 | Stage III | T3 | M0 | N3 | 7th |
| TCGA-BR-8368-01A-11R-23 | FEMALE | G3 | Stage I | T2 | M0 | N0 | 7th |
| TCGA-BR-8369-01A-11R-23 | FEMALE | G3 | Stage III | T3 | M0 | N3 | 7th |
| TCGA-BR-8371-01A-11R-23 | MALE | G3 | Stage III | T3 | M0 | N3 | 7th |
| TCGA-BR-8372-01A-11R-23 | MALE | G3 | Stage III | T4 | M0 | N3 | 7th |
| TCGA-BR-8373-01A-11R-23 | FEMALE | G2 | Stage III | T4 | M0 | N1 | 7th |
| TCGA-BR-8380-01A-11R-23 | MALE | G3 | Stage III | T4 | M0 | N3 | 7th |
| TCGA-BR-8381-01A-11R-24 | MALE | G3 | Stage II | T3 | M0 | N1 | 7th |
| TCGA-BR-8382-01A-11R-24 | FEMALE | G3 | Stage III | T4 | M0 | N3 | 7th |
| TCGA-BR-8384-01A-21R-24 | MALE | G3 | Stage III | T4 | M0 | N2 | 7th |
| TCGA-BR-8483-01A-31R-24 | MALE | G2 | Stage III | T3 | M0 | N2 | 7th |
| TCGA-BR-8484-01A-11R-24 | MALE | G2 | Stage III | T4 | M0 | N1 | 7th |
| TCGA-BR-8485-01A-11R-24 | FEMALE | G3 | Stage III | T4 | M0 | N3 | 7th |
| TCGA-BR-8486-01A-31R-24 | FEMALE | G2 | Stage I | T1 | M0 | N0 | 7th |
| TCGA-BR-8487-01A-11R-24 | FEMALE | G3 | Stage II | T3 | M0 | N0 | 7th |
| TCGA-BR-8588-01A-11R-24 | FEMALE | G3 | Stage II | T4 | M0 | N0 | 7th |
| TCGA-BR-8589-01A-11R-24 | MALE | G3 | Stage III | T4 | M0 | N1 | 7th |
| TCGA-BR-8590-01A-11R-24 | MALE | G3 | Stage III | T4 | M0 | N2 | 7th |
| TCGA-BR-8591-01A-11R-24 | MALE | G3 | Stage III | T4 | M0 | N3 | 7th |
| TCGA-BR-8592-01A-11R-24 | FEMALE | G3 | Stage III | T4 | M0 | N1 | 7th |
| TCGA-BR-8676-01A-11R-24 | MALE | G3 | Stage III | T3 | M0 | N3 | 7th |
| TCGA-BR-8677-01A-11R-24 | FEMALE | G3 | Stage III | T3 | M0 | N3 | 7th |
| TCGA-BR-8678-01A-11R-24 | MALE | G2 | Stage I | T2 | M0 | N0 | 7th |
| TCGA-BR-8679-01A-11R-24 | FEMALE | G2 | Stage I | T2 | M0 | N0 | 7th |
| TCGA-BR-8680-01A-11R-24 | MALE | G2 | Stage IV | T4 | M1 | N2 | 7th |
| TCGA-BR-8682-01A-11R-24 | MALE | G3 | Stage II | T4 | M0 | N0 | 7th |
| TCGA-BR-8683-01A-11R-24 | MALE | G3 | Stage III | T4 | M0 | N2 | 7th |
| TCGA-BR-8686-01A-11R-24 | MALE | G3 | Stage III | T4 | M0 | N1 | 7th |
| TCGA-BR-8687-01A-11R-24 | FEMALE | G3 | Stage III | T4 | M0 | N2 | 7th |
| TCGA-BR-8690-01A-11R-24 | FEMALE | G2 | Stage III | T3 | M0 | N3 | 7th |
| TCGA-BR-A44T-01A-32R-A | FEMALE | G3 | Stage II | T3 | M0 | N0 | 7th |
| TCGA-BR-A44U-01A-11R-A | MALE | G3 | Stage III | T3 | M0 | N3 | 7th |
| TCGA-BR-A4CR-01A-11R-A | FEMALE | G3 | Stage III | T4 | M0 | N3 | 7th |
| TCGA-BR-A4CS-01A-11R-A | MALE | G3 | Stage III | T4 | M0 | N3 | 7th |
| TCGA-BR-A4IV-01A-31R-A2 | MALE | G3 | Stage III | T4 | M0 | N2 | 7th |
| TCGA-BR-A4J4-01A-12R-A2 | MALE | G3 | Stage III | T4 | M0 | N2 | 7th |
| TCGA-BR-A4J5-01A-21R-A2 | MALE | G3 | Stage III | T4 | M0 | N1 | 7th |
| TCGA-BR-A4J6-01A-11R-A2 | FEMALE | G2 | Stage II | T3 | M0 | N0 | 7th |
| TCGA-BR-A4J8-01A-11R-A2 | FEMALE | G3 | Stage III | T3 | M0 | N3 | 7th |
| TCGA-BR-A4J9-01A-12R-A2 | MALE | G3 | Stage II | T3 | M0 | N0 | 7th |
| TCGA-BR-A4PF-01A-11R-A | MALE | G3 | Stage III | T4 | M0 | N2 | 7th |
| TCGA-BR-A4QL-01A-31R-A | FEMALE | G2 | Stage III | T3 | M0 | N3 | 7th |
| TCGA-CD-5798-01A-11R-1 | MALE | G2 | Stage II | T3 | M0 | N0 | 7th |
| TCGA-CD-5799-01A-11R-1 | MALE | G3 | Stage II | T2 | M0 | N1 | 7th |
| TCGA-CD-5800-01A-11R-1 | FEMALE | G1 | Stage II | T3 | M0 | N0 | 7th |
| TCGA-CD-5801-01A-11R-1 | MALE | G3 | Stage II | T3 | M0 | N1 | 7th |
| TCGA-CD-5803-01A-11R-1 | FEMALE | G3 | Stage II | T3 | M0 | N0 | 7th |
| TCGA-CD-5813-01A-11R-1 | MALE | G3 | Stage II | T3 | M0 | N0 | 7th |
| TCGA-CD-8524-01A-11R-2 | FEMALE | G3 | Stage II | T3 | M0 | N0 | 7th |
| TCGA-CD-8525-01A-11R-2 | FEMALE | G3 | Stage II | T3 | M0 | N1 | 7th |
| TCGA-CD-8526-01A-11R-2 | FEMALE | G3 | Stage II | T3 | M0 | N1 | 7th |
| TCGA-CD-8527-01A-11R-2 | FEMALE | G2 | Stage II | T2 | M0 | N1 | 7th |
| TCGA-CD-8528-01A-11R-2 | FEMALE | G3 | Stage III | T4 | M0 | N0 | 7th |
| TCGA-CD-8529-01A-11R-2 | MALE | G2 | Stage IV | T4 | M1 | N0 | 7th |
| TCGA-CD-8530-01A-11R-2 | MALE | G3 | Stage II | T3 | M0 | N0 | 7th |
| TCGA-CD-8531-01A-11R-2 | FEMALE | G3 | Stage II | T3 | M0 | N1 | 7th |
| TCGA-CD-8532-01A-11R-2 | MALE | G2 | Stage II | T3 | M0 | N0 | 7th |
| TCGA-CD-8533-01A-11R-2 | MALE | G3 | Stage II | T3 | M0 | N0 | 7th |
| TCGA-CD-8534-01A-11R-2 | MALE | G3 | Stage II | T3 | M0 | N0 | 7th |
| TCGA-CD-8535-01A-11R-2 | MALE | G3 | Stage III | T3 | M0 | N2 | 7th |
| TCGA-CD-A486-01A-11R-A | MALE | G2 | Stage II | T3 | M0 | N0 | 7th |
| TCGA-CD-A487-01A-21R-A | MALE | G3 | Stage II | T3 | M0 | N1 | 7th |
| TCGA-CD-A489-01A-11R-A | MALE | G3 | Stage II | T3 | M0 | N0 | 7th |
| TCGA-CD-A48A-01A-12R-A | MALE | G2 | Stage II | T3 | M0 | N0 | 7th |
| TCGA-CD-A48C-01A-11R-A | FEMALE | G3 | Stage II | T3 | M0 | N1 | 7th |
| TCGA-CD-A4MG-01A-11R- | MALE | G3 | Stage II | T3 | M0 | N0 | 7th |
| TCGA-CD-A4MH-01A-11R- | FEMALE | G2 | Stage II | T3 | M0 | N0 | 7th |
| TCGA-CG-4301-01A-01R-1 | FEMALE | G3 | Stage IV | T4 | M1 | N2 | 7th |
| TCGA-CG-4304-01A-01R-1 | MALE | G2 | Stage I | T2 | M0 | N0 | 7th |
| TCGA-CG-4305-01A-01R-1 | MALE | G3 | Stage II | T2 | M0 | N1 | 7th |
| TCGA-CG-4306-01A-01R-1 | MALE | G3 | Stage IV | T3 | M1 | N0 | 7th |
| TCGA-CG-4436-01A-01R-1 | MALE | G2 | Stage I | T2 | M0 | N0 | 7th |
| TCGA-CG-4437-01A-01R-1 | MALE | G2 | Stage II | T2 | M0 | N2 | 7th |
| TCGA-CG-4438-01A-01R-1 | MALE | G3 | Stage III | T4 | M0 | N3 | 7th |
| TCGA-CG-4440-01A-01R-1 | FEMALE | G3 | Stage IV | T3 | M1 | N3 | 7th |
| TCGA-CG-4441-01A-01R-1 | MALE | G2 | Stage III | T2 | M0 | N3 | 7th |
| TCGA-CG-4442-01A-01R-1 | MALE | G3 | Stage I | T2 | M0 | N0 | 7th |
| TCGA-CG-4443-01A-01R-1 | MALE | G2 | Stage I | T1 | M0 | N0 | 7th |
| TCGA-CG-4444-01A-01R-1 | MALE | G3 | Stage III | T3 | M0 | N3 | 7th |
| TCGA-CG-4460-01A-01R-1 | FEMALE | G2 | Stage IV | T4 | M1 | N1 | 7th |
| TCGA-CG-4462-01A-01R-1 | FEMALE | G3 | Stage IV | T3 | M1 | N3 | 7th |
| TCGA-CG-4465-01A-01R-1 | FEMALE | G3 | Stage IV | T4 | M1 | N3 | 7th |
| TCGA-CG-4466-01A-01R-1 | FEMALE | G2 | Stage I | T2 | M0 | N0 | 7th |
| TCGA-CG-4469-01A-01R-1 | MALE | G3 | Stage III | T3 | M0 | N3 | 7th |
| TCGA-CG-4475-01A-01R-1 | MALE | G3 | Stage II | T3 | M0 | N1 | 7th |
| TCGA-CG-4476-01A-01R-1 | MALE | G3 | Stage III | T4 | M0 | N3 | 7th |
| TCGA-CG-4477-01A-01R-1 | FEMALE | G3 | Stage I | T2 | M0 | N0 | 7th |
| TCGA-CG-5716-01A-21R-1 | MALE | G2 | Stage IV | T4 | M1 | N2 | 7th |
| TCGA-CG-5717-01A-11R-1 | MALE | G3 | Stage III | T3 | M0 | N2 | 7th |
| TCGA-CG-5718-01A-11R-1 | FEMALE | G2 | Stage III | T3 | M0 | N2 | 7th |
| TCGA-CG-5719-01A-11R-1 | FEMALE | G1 | Stage IV | T4 | M1 | N0 | 7th |
| TCGA-CG-5720-01A-11R-1 | MALE | G3 | Stage I | T2 | M0 | N0 | 7th |
| TCGA-CG-5721-01A-11R-1 | MALE | G3 | Stage III | T4 | M0 | N1 | 7th |
| TCGA-CG-5722-01A-21R-1 | FEMALE | G3 | Stage IV | T3 | M1 | N3 | 7th |
| TCGA-CG-5723-01A-11R-1 | MALE | G3 | Stage II | T2 | M0 | N1 | 7th |
| TCGA-CG-5724-01A-11R-1 | MALE | G3 | Stage IV | T3 | M1 | N3 | 7th |
| TCGA-CG-5725-01A-11R-1 | MALE | G3 | Stage I | T2 | M0 | N0 | 7th |
| TCGA-CG-5726-01A-11R-1 | MALE | G2 | Stage I | T1 | M0 | N0 | 7th |
| TCGA-CG-5732-01A-11R-1 | MALE | G2 | Stage III | T2 | M0 | N3 | 7th |
| TCGA-CG-5734-01A-11R-1 | MALE | G3 | Stage III | T3 | M0 | N2 | 7th |
| TCGA-D7-5577-01A-01R-1 | FEMALE | G2 | Stage III | T2 | M0 | N3 | 7th |
| TCGA-D7-5578-01A-01R-1 | MALE | G3 | Stage III | T3 | M0 | N2 | 7th |
| TCGA-D7-6520-01A-11R-1 | MALE | G3 | Stage III | T3 | M0 | N3 | 7th |
| TCGA-D7-6522-01A-11R-1 | MALE | G3 | Stage I | T2 | M0 | N0 | 7th |
| TCGA-D7-6524-01A-11R-1 | MALE | G3 | Stage II | T2 | M0 | N2 | 7th |
| TCGA-D7-6525-01A-11R-1 | MALE | G3 | Stage II | T2 | M0 | N2 | 7th |
| TCGA-D7-6526-01A-11R-1 | FEMALE | G2 | Stage III | T3 | M0 | N2 | 7th |
| TCGA-D7-6527-01A-11R-1 | MALE | G2 | Stage II | T2 | M0 | N1 | 7th |
| TCGA-D7-6528-01A-11R-1 | FEMALE | G2 | Stage I | T2 | M0 | N0 | 7th |
| TCGA-D7-6815-01A-11R-1 | FEMALE | G2 | Stage II | T2 | M0 | N2 | 7th |
| TCGA-D7-6818-01A-11R-1 | MALE | G3 | Stage III | T2 | M0 | N3 | 7th |
| TCGA-D7-6822-01A-11R-1 | MALE | G2 | Stage I | T2 | M0 | N0 | 7th |
| TCGA-D7-8570-01A-11R-2 | MALE | G3 | Stage III | T3 | M0 | N3 | 7th |
| TCGA-D7-8572-01A-11R-2 | MALE | G2 | Stage II | T2 | M0 | N2 | 7th |
| TCGA-D7-8573-01A-11R-2 | MALE | G3 | Stage II | T3 | M0 | N0 | 7th |
| TCGA-D7-8574-01A-13R-2 | MALE | G3 | Stage III | T2 | M0 | N3 | 7th |
| TCGA-D7-8575-01A-11R-2 | MALE | G3 | Stage III | T3 | M0 | N2 | 7th |
| TCGA-D7-8576-01A-11R-2 | FEMALE | G3 | Stage III | T3 | M0 | N3 | 7th |
| TCGA-D7-8578-01A-21R-2 | MALE | G2 | Stage I | T2 | M0 | N0 | 7th |
| TCGA-D7-8579-01A-11R-2 | FEMALE | G2 | Stage II | T2 | M0 | N2 | 7th |
| TCGA-D7-A4YU-01A-21R-A | MALE | G3 | Stage III | T3 | M0 | N3 | 7th |
| TCGA-D7-A4YX-01A-11R-A | MALE | G3 | Stage II | T3 | M0 | N1 | 7th |
| TCGA-D7-A4Z0-01A-22R-A | FEMALE | G3 | Stage II | T2 | M0 | N2 | 7th |
| TCGA-D7-A6EV-01A-11R-A | FEMALE | G3 | Stage II | T2 | M0 | N2 | 7th |
| TCGA-D7-A6EX-01A-11R-A | FEMALE | G3 | Stage III | T3 | M0 | N2 | 7th |
| TCGA-D7-A6EY-01A-21R-A | FEMALE | G3 | Stage III | T3 | M0 | N3 | 7th |
| TCGA-D7-A6EZ-01A-11R-A | MALE | G3 | Stage III | T3 | M0 | N2 | 7th |
| TCGA-D7-A6F0-01A-11R-A | FEMALE | G3 | Stage I | T2 | M0 | N0 | 7th |
| TCGA-D7-A6F2-01A-12R-A | MALE | G3 | Stage I | T2 | M0 | N0 | 7th |
| TCGA-D7-A747-01A-22R-A | MALE | G3 | Stage II | T3 | M0 | N1 | 7th |
| TCGA-D7-A748-01A-12R-A | FEMALE | G3 | Stage IV | T4 | M1 | N3 | 7th |
| TCGA-D7-A74A-01A-11R-A | FEMALE | G2 | Stage III | T3 | M0 | N2 | 7th |
| TCGA-F1-6874-01A-11R-18 | MALE | G3 | Stage I | T2 | M0 | N0 | 7th |
| TCGA-F1-6875-01A-11R-20 | MALE | G2 | Stage I | T2 | M0 | N0 | 7th |
| TCGA-F1-A448-01A-11R-A | MALE | G3 | Stage III | T3 | M0 | N3 | 7th |
| TCGA-F1-A72C-01A-21R-A | MALE | G2 | Stage II | T3 | M0 | N0 | 7th |
| TCGA-FP-7829-01A-11R-20 | MALE | G2 | Stage II | T3 | M0 | N1 | 7th |
| TCGA-FP-8209-01A-11R-23 | MALE | G3 | Stage I | T2 | M0 | N0 | 7th |
| TCGA-FP-8210-01A-11R-23 | MALE | G3 | Stage III | T3 | M0 | N2 | 7th |
| TCGA-FP-A4BF-01A-12R-A | MALE | G3 | Stage III | T3 | M0 | N2 | 7th |
| TCGA-HF-7132-01A-11R-2 | MALE | G2 | Stage II | T3 | M0 | N1 | 7th |
| TCGA-HF-7133-01A-11R-2 | FEMALE | G2 | Stage III | T3 | M0 | N3 | 7th |
| TCGA-HF-7134-01A-11R-2 | MALE | G2 | Stage I | T1 | M0 | N0 | 7th |
| TCGA-HF-A5NB-01A-11R-A | FEMALE | G3 | Stage III | T4 | M0 | N3 | 7th |
| TCGA-HU-8244-01A-11R-2 | FEMALE | G1 | Stage I | T1 | M0 | N0 | 7th |
| TCGA-HU-8249-01A-11R-A | MALE | G2 | Stage III | T3 | M0 | N2 | 7th |
| TCGA-HU-8602-01A-11R-2 | FEMALE | G3 | Stage II | T4 | M0 | N0 | 7th |
| TCGA-HU-8604-01A-11R-2 | FEMALE | G2 | Stage II | T3 | M0 | N0 | 7th |
| TCGA-HU-8608-01A-11R-2 | MALE | G3 | Stage III | T4 | M0 | N2 | 7th |
| TCGA-HU-8610-01A-22R-2 | MALE | G2 | Stage I | T1 | M0 | N0 | 7th |
| TCGA-HU-A4G2-01A-11R- | MALE | G3 | Stage II | T3 | M0 | N1 | 7th |
| TCGA-HU-A4G3-01A-11R- | MALE | G2 | Stage II | T2 | M0 | N2 | 7th |
| TCGA-HU-A4G8-01A-11R- | FEMALE | G3 | Stage II | T3 | M0 | N1 | 7th |
| TCGA-HU-A4G9-01A-11R- | FEMALE | G2 | Stage I | T1 | M0 | N0 | 7th |
| TCGA-HU-A4GC-01A-12R- | MALE | G3 | Stage III | T4 | M0 | N2 | 7th |
| TCGA-HU-A4GD-01A-11R- | MALE | G3 | Stage II | T3 | M0 | N1 | 7th |
| TCGA-HU-A4GF-01A-11R-A | MALE | G2 | Stage II | T3 | M0 | N0 | 7th |
| TCGA-HU-A4GH-01A-11R- | MALE | G2 | Stage I | T1 | M0 | N0 | 7th |
| TCGA-HU-A4GJ-01A-11R-A | FEMALE | G3 | Stage III | T4 | M0 | N3 | 7th |
| TCGA-HU-A4GP-01A-11R- | FEMALE | G2 | Stage II | T2 | M0 | N1 | 7th |
| TCGA-HU-A4GQ-01A-11R- | MALE | G2 | Stage III | T4 | M0 | N2 | 7th |
| TCGA-HU-A4GT-01A-21R- | FEMALE | G3 | Stage II | T3 | M0 | N0 | 7th |
| TCGA-HU-A4GU-01A-11R- | MALE | G3 | Stage II | T3 | M0 | N1 | 7th |
| TCGA-HU-A4GX-01A-12R- | FEMALE | G3 | Stage III | T4 | M0 | N2 | 7th |
| TCGA-HU-A4GY-01A-21R- | FEMALE | G3 | Stage III | T4 | M0 | N1 | 7th |
| TCGA-HU-A4H0-01A-11R- | MALE | G3 | Stage III | T4 | M0 | N3 | 7th |
| TCGA-HU-A4H2-01A-11R- | FEMALE | G2 | Stage III | T3 | M0 | N3 | 7th |
| TCGA-HU-A4H3-01A-21R- | FEMALE | G3 | Stage III | T4 | M0 | N3 | 7th |
| TCGA-HU-A4H4-01A-21R- | FEMALE | G3 | Stage II | T2 | M0 | N2 | 7th |
| TCGA-HU-A4H6-01A-11R- | FEMALE | G2 | Stage III | T3 | M0 | N2 | 7th |
| TCGA-HU-A4H8-01A-11R- | MALE | G2 | Stage I | T1 | M0 | N1 | 7th |
| TCGA-HU-A4HB-01A-12R- | MALE | G3 | Stage II | T2 | M0 | N2 | 7th |
| TCGA-HU-A4HD-01A-11R- | MALE | G2 | Stage III | T3 | M0 | N2 | 7th |
| TCGA-IN-7806-01A-11R-20 | MALE | G3 | Stage II | T3 | M0 | N1 | 7th |
| TCGA-IN-8462-01A-11R-23 | MALE | G2 | Stage II | T2 | M0 | N1 | 7th |
| TCGA-IN-8663-01A-11R-24 | MALE | G3 | Stage II | T2 | M0 | N2 | 7th |
| TCGA-IN-A6RI-01A-11R-A3 | MALE | G2 | Stage I | T1 | M0 | N0 | 7th |
| TCGA-IN-A6RJ-01A-21R-A3 | MALE | G3 | Stage I | T1 | M0 | N0 | 7th |
| TCGA-IN-A6RN-01A-12R-A | FEMALE | G2 | Stage II | T1 | M0 | N2 | 7th |
| TCGA-IN-A6RO-01A-12R-A | MALE | G2 | Stage I | T1 | M0 | N0 | 7th |
| TCGA-IN-A6RS-01A-12R-A3 | MALE | G2 | Stage I | T1 | M0 | N0 | 7th |
| TCGA-IN-A7NR-01A-11R-A | FEMALE | G3 | Stage IV | T3 | M1 | N3 | 7th |
| TCGA-IN-A7NT-01A-21R-A | FEMALE | G3 | Stage II | T3 | M0 | N1 | 7th |
| TCGA-IN-A7NU-01A-22R-A | MALE | G3 | Stage III | T3 | M0 | N3 | 7th |
| TCGA-IN-AB1V-01A-21R-A | MALE | G2 | Stage I | T1 | M0 | N0 | 7th |
| TCGA-IN-AB1X-01A-11R-A3 | FEMALE | G3 | Stage II | T3 | M0 | N0 | 7th |
| TCGA-KB-A6F7-01A-12R-A3 | FEMALE | G3 | Stage I | T1 | M0 | N1 | 7th |
| TCGA-KB-A93G-01A-11R-A | MALE | G1 | Stage I | T2 | M0 | N0 | 7th |
| TCGA-KB-A93H-01A-11R-A | FEMALE | G1 | Stage II | T3 | M0 | N1 | 7th |
| TCGA-KB-A93J-01A-11R-A3 | MALE | G3 | Stage II | T3 | M0 | N1 | 7th |
| TCGA-MX-A5UG-01A-21R- | MALE | G3 | Stage II | T3 | M0 | N1 | 7th |
| TCGA-MX-A5UJ-01A-11R-A | FEMALE | G3 | Stage III | T3 | M0 | N2 | 7th |
| TCGA-MX-A663-01A-11R-A | MALE | G3 | Stage II | T3 | M0 | N0 | 7th |
| TCGA-R5-A7O7-01A-11R-A | MALE | G2 | Stage IV | T3 | M1 | N1 | 7th |
| TCGA-R5-A7ZE-01B-11R-A3 | FEMALE | G2 | Stage III | T3 | M0 | N2 | 7th |
| TCGA-R5-A7ZF-01A-11R-A3 | FEMALE | G2 | Stage IV | T4 | M1 | N1 | 7th |
| TCGA-R5-A7ZI-01A-11R-A3 | FEMALE | G3 | Stage IV | T4 | M1 | N1 | 7th |
| TCGA-R5-A805-01A-11R-A3 | MALE | G3 | Stage III | T3 | M0 | N3 | 7th |
| TCGA-RD-A7BS-01A-11R-A | MALE | G3 | Stage III | T3 | M0 | N2 | 7th |
| TCGA-RD-A7BT-01A-11R-A | MALE | G3 | Stage III | T3 | M0 | N3 | 7th |
| TCGA-RD-A7BW-01A-11R- | FEMALE | G3 | Stage I | T2 | M0 | N0 | 7th |
| TCGA-RD-A7C1-01A-11R-A | MALE | G3 | Stage I | T2 | M0 | N0 | 7th |
| TCGA-RD-A8MV-01A-11R- | MALE | G3 | Stage III | T3 | M0 | N3 | 7th |
| TCGA-RD-A8MW-01A-11R- | MALE | G3 | Stage III | T3 | M0 | N3 | 7th |
| TCGA-RD-A8N0-01A-12R-A | FEMALE | G3 | Stage III | T3 | M0 | N3 | 7th |
| TCGA-RD-A8N1-01A-12R-A | MALE | G3 | Stage III | T3 | M0 | N3 | 7th |
| TCGA-RD-A8N2-01A-12R-A | FEMALE | G3 | Stage I | T2 | M0 | N0 | 7th |
| TCGA-RD-A8N4-01A-21R-A | FEMALE | G3 | Stage III | T3 | M0 | N2 | 7th |
| TCGA-RD-A8N5-01A-12R-A | MALE | G2 | Stage III | T3 | M0 | N2 | 7th |
| TCGA-RD-A8N6-01A-11R-A | FEMALE | G3 | Stage III | T2 | M0 | N3 | 7th |
| TCGA-RD-A8N9-01A-12R-A | FEMALE | G3 | Stage II | T2 | M0 | N1 | 7th |
| TCGA-RD-A8NB-01A-12R-A | FEMALE | G3 | Stage III | T3 | M0 | N2 | 7th |
| TCGA-SW-A7EB-01A-11R-A | MALE | G2 | Stage III | T3 | M0 | N2 | 7th |
| TCGA-VQ-A8DT-01A-11R-A | MALE | G3 | Stage III | T3 | M0 | N3 | 7th |
| TCGA-VQ-A8DU-01A-11R- | MALE | G2 | Stage III | T3 | M0 | N2 | 7th |
| TCGA-VQ-A8DV-01A-12R- | MALE | G2 | Stage I | T2 | M0 | N0 | 7th |
| TCGA-VQ-A8DZ-01A-11R-A | MALE | G3 | Stage IV | T4 | M1 | N3 | 7th |
| TCGA-VQ-A8E0-01A-11R-A | MALE | G3 | Stage III | T3 | M0 | N2 | 7th |
| TCGA-VQ-A8E2-01A-11R-A | MALE | G2 | Stage III | T3 | M0 | N3 | 7th |
| TCGA-VQ-A8E3-01A-11R-A | MALE | G3 | Stage II | T3 | M0 | N0 | 7th |
| TCGA-VQ-A8E7-01B-11R-A | MALE | G2 | Stage IV | T3 | M1 | N1 | 7th |
| TCGA-VQ-A8P2-01A-11R-A | MALE | G2 | Stage III | T4 | M0 | N1 | 7th |
| TCGA-VQ-A8P3-01A-11R-A | MALE | G2 | Stage III | T4 | M0 | N1 | 7th |
| TCGA-VQ-A8P5-01A-11R-A | MALE | G3 | Stage II | T3 | M0 | N0 | 7th |
| TCGA-VQ-A8P8-01A-11R-A | FEMALE | G2 | Stage II | T4 | M0 | N0 | 7th |
| TCGA-VQ-A8PB-01A-11R-A | FEMALE | G2 | Stage II | T3 | M0 | N0 | 7th |
| TCGA-VQ-A8PC-01A-11R-A | MALE | G3 | Stage II | T3 | M0 | N1 | 7th |
| TCGA-VQ-A8PD-01A-11R-A | MALE | G2 | Stage III | T4 | M0 | N3 | 7th |
| TCGA-VQ-A8PE-01A-11R-A | MALE | G3 | Stage III | T3 | M0 | N3 | 7th |
| TCGA-VQ-A8PF-01A-11R-A | MALE | G3 | Stage III | T3 | M0 | N3 | 7th |
| TCGA-VQ-A8PH-01A-12R-A | MALE | G2 | Stage III | T3 | M0 | N3 | 7th |
| TCGA-VQ-A8PK-01A-12R-A | MALE | G2 | Stage III | T3 | M0 | N3 | 7th |
| TCGA-VQ-A8PM-01A-21R- | MALE | G3 | Stage III | T4 | M0 | N3 | 7th |
| TCGA-VQ-A8PO-01A-11R- | MALE | G3 | Stage II | T4 | M0 | N0 | 7th |
| TCGA-VQ-A8PP-01A-21R-A | MALE | G2 | Stage III | T4 | M0 | N2 | 7th |
| TCGA-VQ-A8PQ-01A-11R- | FEMALE | G2 | Stage IV | T4 | M1 | N2 | 7th |
| TCGA-VQ-A8PU-01A-12R-A | FEMALE | G3 | Stage III | T4 | M0 | N1 | 7th |
| TCGA-VQ-A8PX-01A-12R-A | MALE | G2 | Stage I | T1 | M0 | N0 | 7th |
| TCGA-VQ-A91A-01A-11R-A | MALE | G1 | Stage III | T3 | M0 | N3 | 7th |
| TCGA-VQ-A91D-01A-11R-A | MALE | G2 | Stage III | T4 | M0 | N2 | 7th |
| TCGA-VQ-A91E-01A-31R-A | FEMALE | G3 | Stage III | T4 | M0 | N0 | 7th |
| TCGA-VQ-A91K-01A-11R-A | MALE | G2 | Stage III | T3 | M0 | N2 | 7th |
| TCGA-VQ-A91N-01A-11R-A | FEMALE | G2 | Stage III | T4 | M0 | N3 | 7th |
| TCGA-VQ-A91Q-01A-12R- | MALE | G3 | Stage III | T3 | M0 | N3 | 7th |
| TCGA-VQ-A91U-01A-11R-A | MALE | G2 | Stage II | T3 | M0 | N1 | 7th |
| TCGA-VQ-A91V-01A-11R-A | MALE | G2 | Stage III | T3 | M0 | N2 | 7th |
| TCGA-VQ-A91X-01A-12R-A | MALE | G3 | Stage III | T3 | M0 | N3 | 7th |
| TCGA-VQ-A91Y-01A-11R-A | MALE | G3 | Stage III | T4 | M0 | N3 | 7th |
| TCGA-VQ-A91Z-01A-11R-A | FEMALE | G2 | Stage II | T3 | M0 | N1 | 7th |
| TCGA-VQ-A922-01A-11R-A | MALE | G2 | Stage III | T4 | M0 | N1 | 7th |
| TCGA-VQ-A923-01A-11R-A | MALE | G3 | Stage III | T3 | M0 | N2 | 7th |
| TCGA-VQ-A924-01A-11R-A | MALE | G2 | Stage II | T3 | M0 | N0 | 7th |
| TCGA-VQ-A925-01A-11R-A | MALE | G2 | Stage III | T3 | M0 | N2 | 7th |
| TCGA-VQ-A927-01A-12R-A | MALE | G1 | Stage III | T3 | M0 | N3 | 7th |
| TCGA-VQ-A928-01A-11R-A | MALE | G2 | Stage III | T3 | M0 | N3 | 7th |
| TCGA-VQ-A92D-01A-11R-A | MALE | G2 | Stage II | T3 | M0 | N0 | 7th |
| TCGA-VQ-A94O-01A-11R- | MALE | G2 | Stage III | T4 | M0 | N3 | 7th |
| TCGA-VQ-A94T-01A-11R-A | MALE | G3 | Stage III | T3 | M0 | N3 | 7th |
| TCGA-VQ-A94U-01A-12R-A | MALE | G3 | Stage II | T4 | M0 | N0 | 7th |
| TCGA-VQ-AA64-01A-11R-A | MALE | G2 | Stage III | T3 | M0 | N2 | 7th |
| TCGA-VQ-AA68-01A-11R-A | FEMALE | G2 | Stage III | T4 | M0 | N3 | 7th |
| TCGA-VQ-AA69-01A-11R-A | MALE | G2 | Stage III | T3 | M0 | N2 | 7th |
| TCGA-VQ-AA6A-01A-11R- | MALE | G2 | Stage III | T4 | M0 | N3 | 7th |
| TCGA-VQ-AA6D-01A-11R- | FEMALE | G2 | Stage III | T4 | M0 | N1 | 7th |
| TCGA-VQ-AA6F-01A-31R-A | MALE | G2 | Stage II | T3 | M0 | N1 | 7th |
| TCGA-VQ-AA6G-01A-11R- | MALE | G2 | Stage II | T3 | M0 | N0 | 7th |
| TCGA-VQ-AA6J-01A-11R-A | MALE | G3 | Stage III | T4 | M0 | N2 | 7th |
| TCGA-VQ-AA6K-01A-11R-A | MALE | G3 | Stage III | T4 | M0 | N3 | 7th |

| Primer name | Sequence (5' to 3') |
|---|---|
| GAPDH-Forward | GGAGCGAGATCCCTCCAAAAT |
| GAPDH-Reverse | GGCTGTTGTCATACTTCTCATGG |
| CPNE8-Forward | GAATGGAGAGAGTTTGGAAGA |
| CPNE8-Reverse | GTCATGTTTGGATAAGTTGGG |
| PTK2(FAK)-Forward | CCAGGTCAGGCATCTCTT |
| PTK2(FAK)-Reverse | CCTCGCAGGTCCAATACT |
| ERK-Forward | TACACCAACCTCTCGTACATCG |
| ERK-Reverse | CATGTCTGAAGCGCAGTAAGATT |
| CCR4-Forward | GCTCCCTGGAAATCAACA |
| CCR4-Reverse | TCATCTTCACCGCCTTG |
| CXCR4-Forward | ACTACACCGAGGAAATGGGCT |
| CXCR4-Reverse | CCCACAATGCCAGTTAAGAAGA |
